# HOXC8 impacts lung tumorigenesis by preventing pyroptotic cell death through the suppression of caspase-1 expression

**DOI:** 10.1038/s41419-025-07867-8

**Published:** 2025-07-23

**Authors:** Ravi Padia, Lei Sun, Ya-Fang Liao, Ozlem Calbay, Cheng Chi, Mahmuda Akter, Lizi Wu, Zheng Fu, Dan-Dan Zhang, Shuang Huang

**Affiliations:** 1 Department of Anatomy and Cell Biology, University of Florida College of Medicine, Gainesville, FL USA; 2 Institute of Interdisciplinary Integrative Medicine Research, Shanghai University of Traditional Chinese Medicine, Shanghai, China; 3 Department of Molecular Genetics and Microbiology, University of Florida College of Medicine, Gainesville, FL USA; 4 Department of Human and Molecular Genetics, Virginia Commonwealth University, School of Medicine, Richmond, VA USA

**Keywords:** Cancer epigenetics, Cancer epigenetics

## Abstract

Homeobox C8 (HOXC8) is a transcription factor preferentially overexpressed in a large percentage of non-small cell lung carcinoma (NSCLC). To investigate the function of HOXC8 in NSCLC, we showed that knockdown of HOXC8 led to massive NSCLC cell death in a mechanism of pyroptosis because both YVAD, a caspase-1 (CASP1) inhibitor, and disulfiram, which prevents gasdermin D (GSDMD) pore formation, blocked cell death caused by HOXC8 depletion. Intriguingly, ASC, a key component of canonic inflammasome, was dispensable for pyroptosis occurring in HOXC8-depleted cells. Instead, we detected greatly elevated levels of both CASP1 protein and mRNA in HOXC8-knockdown cells. As forced expression of CASP1 is sufficient to induce CASP1 activation and pyroptosis, we reason that pyroptosis led by HOXC8 depletion results from massive increase in the abundance of CASP1. To uncover the functional connection between HOXC8 and CASP1 expression, we revealed that HDAC1/2 was involved in augmented CASP1 transcription induced by HOXC8 knockdown. Moreover, we found that HOXC8 and HDAC1 were in the same immunocomplex and the presence of HOXC8 is required for the recruitment of HDAC1 to CASP1 promoter. Since HOXC8 also binds CASP1 promoter, we conclude that HOXC8 negatively regulates CASP1 expression by drafting HDAC1/2 to CASP1 gene. Finally, we demonstrated that cholesterol-conjugated HOXC8 siRNA was able to slow down NSCLC tumorigenesis. This study suggests that HOXC8 participates NSCLC development by controlling CASP1 expression and pyroptosis.

## Introduction

*HOXC8* belongs to highly conserved subgroup of 39 genes housed into four clusters *HOXA-HOXD* within the superfamily of homeobox genes. *HOX* genes encode homeodomain- transcription factors that are responsible for regulating morphogenesis through axial patterning during embryogenesis. In addition to its well established developmental role, dysregulated HOXC8 expression has been observed in various cancer types. In Glioma, upregulated HOXC8 promotes tumorigenesis via its transcriptional target MDM2 which then blunts the tumor suppressive effects of p53 [1]. Primary prostate tumors and cell lines exhibit higher HOXC8 levels that correlate with higher androgen-independent cell proliferation and loss of differentiation [2]. HOXC8 is highly expressed in clinical specimens of cervical cancer and is associated with poor prognosis [3]. Previously, we have shown that HOXC8 acts as a transcriptional activator to promote expression of CDH11 in basal-like breast cancer cells leading to increased anchorage-independent cell growth, migration and invasion [4]. Another study reported that HOXC8 promotes breast tumorigenesis by transcriptionally repressing tumor suppressor Embigin [5]. Given the spatiotemporal expression of HOXC8, it may also act as a tumor suppressor depending on the organ involved. For example, HOXC8 expression is inversely correlated to progression of pancreatic ductal adenocarcinoma primary tumors. Depletion of HOXC8 led to increased cell proliferation, colony formation and migration in pancreatic adenocarcinoma cell lines [6]. It would be of great interest to systematically analyze HOXC8 expression in various cancer types to better ascertain the role of HOXC8 in tumorigenesis.

Pyroptosis is a pro-inflammatory programmed cell death first observed in macrophages and has since been studied extensively in the context of immune system [7]. Pyroptosis is effected mainly by two different types of protein complexes—canonical and non-canonical inflammosomes. In canonical inflammosome, external pathogen or endogenous damage induce oligomerization of sensor proteins like NLRP3, NLRC4, AIM2, or Pyrin to form complex with adapter protein ASC which then provides platform for pro-caspase1 to oligomerize resulting into proximity induced procaspase-1 self-cleavage and activation [8]. Activated caspase-1 cleaves GSDMD which compromises membrane integrity by forming pores in the plasma membrane ultimately inflicting cellular death [9]. Contrarily, in the non-canonical pyroptosis, cytosolic lipopolysaccharide (LPS) is directly recognized by pro-caspase 4/5 or pro-caspase 11 (murine) eliciting caspase-oligomerization and activation leading to the cleavage of GSDMD [10]. Recent studies suggest that pyroptosis can play both tumor-inhibiting and promoting role depending on the context. Pyroptosis mediated through NLRP3/IL-1β axis in macrophages was reported to facilitate obesity-associated pancreatic cancer progression [11]. However, α-NETA or citric acid was found to inhibit epithelial ovarian cancer cell growth by inducing caspase-4/GSDMD-mediated pyroptosis [12, 13]. We recently demonstrated that polyunsaturated fatty acids trigger a caspase-1/GSDMD-mediated pyroptosis in ovarian cancer cells in a mechanism requiring translational augmentation of caspase-1 that is independent of ASC/inflammasome [14]. Nevertheless, it remains to be seen whether ASC/inflammasome-independent but caspase-1-mediated pyroptosis can take place in other epithelial-derived cells.

Through the analysis of HOXC8 expression in various cancer types, we revealed that HOXC8 is preferentially overexpressed in lung adenocarcinoma compared to normal lung tissue. To investigate the potential role of HOXC8 in lung adenocarcinoma, we found that depletion of HOXC8 led to pyroptosis. Surprisingly knockdown of ASC did not prevent cell death induced by HOXC8 depletion in lung cancer cell lines. Interestingly, we observed a striking increase in the abundance of procaspase-1 and its cleavage in HOXC8 depleted cells. Depletion of HOXC8 also increased *CASP1* transcription and decreased the occupancy of HDAC1 to *CASP1* promoter. HOXC8 directly interacts with HDAC1, suggesting that HOXC8 represses *CASP1* transcription by recruiting HDAC1 to the *CASP1* promoter. Bioinformatics analysis revealed that caspase-1 expression is generally lower in lung cancer and is inversely correlated with HOXC8 expression. Our study unravels previously unknown role of HOXC8 that regulate lung tumorigenesis by repressing caspase-1 expression and pyroptosis.

## Materials and methods

### Cell culture, transfection, and Lentivirus preparations

Cells were cultured at 37°C in a humidified chamber with 5% CO_2_ in DMEM supplemented with 10% FBS and 1% Pen/Strep. Transfections on cell lines of interest were performed with Lipofectamine 3000 (Thermo Fisher Scientific, Waltham, MA) as per manufacturer’s instructions. Lentiviruses were prepared in 293FT cells using Virapower lentiviral packaging mix (Thermo Fisher Scientific). A549 and H460 cell lines were infected with the lentivirus of interest according to supplier’s instructions. Mouse macrophage cells were kindly provided by Dr. Nagendra Singh, Augusta University, Augusta, GA.

### Antibodies

Caspase-3 (#14220), RP-II (#2629), HDAC1 (#34589), GST (#2624), ASC (#13833) and β-Actin (#3700, #4970) antibodies were purchased from Cell Signaling Technology (Danvers, MA). Caspase-1 (#22915-1-AP), Gasdermin D (#20770-1-AP) and HOXC8 (#15448-1-AP) antibodies were purchased from Proteintech Group (Rosemont, IL). HA (#sc-7392) and HDAC1 (sc-8410) antibodies were purchased from Santa Cruz Biotechnology (Santa Cruz, CA). ASC (#04-147) used for immunofluorescence was procured from MilliporeSigma (Burlington, MA).

### Plasmids

Plasmid pCI-Caspase-1 (#41522) was obtained from Addgene (Watertown, MA). Caspase-1 D27G mutant was generated from Wild-type Caspase-1 by performing site-directed mutagenesis using QuickChange® site-directed mutagenesis kit (Agilent, Santa Clara, CA). HOXC8 shRNA and HOXC8 expression constructs are as previously described [15]. PYCARD (ASC) shRNAs were obtained from MilliporeSigma’s TRC collection (TRCN0000059073 and TRCN0000059075).

### Reagents

Z-VAD-FMK, Z-YVAD-FMK, Z-VDVAD-FMK, Z-DEVD-FMK, Z-WEHD-FMK, Z-VEID-FMK, Z-IETD-FMK, Z-LEHD-FMK were from APExBIO (Houston, TX). Z-LEVD-FMK was from BioVision (Milpitas, CA). Z-AEVD-FMK and Necrostatin-1 were from R&D Systems (Minneapolis, MN). Chloroquine and MTT were from MilliporeSigma. Romidepsin, TSA and Disulfiram were from Selleck Chemicals (Houston TX).

### Flow cytometry to analyze cell cycle

A549 and H460 cells were infected with lentivirus encoding scrambled sequence or HOXC8 shRNAs. Post 4 days of infection, effect of HOXC8 depletion on cell cycle progression was assessed by Propidium Iodide Flow Cytometry Kit purchased from Abcam (# ab139418) (Boston, MA) and subjected to flow cytometry using BD Accuri C6 Plus flow cytometer (BD Biosciences, Franklin Lakes, NJ). Data were analyzed using the BD FACSDiva Software.

### MTT assay

A549 and H460 cells were first lentivirally transduced with scrambled sequence or HOXC8 shRNAs for 2 days and then seeded at a density of 10,000 cells per well in 24-well plates in the presence of various inhibitors or vehicle (DMSO). Cells were cultured for additional 2 days and cell growth was assessed by adding MTT solution to the cells followed by incubation at 37°C for 1 hour. The MTT formazan crystals generated were dissolved in DMSO, and optical density was measured at 570 nm using a Bio-Rad plate reader.

### Western Blotting

Cells were washed twice with ice-cold PBS and lysed with RIPA buffer (50 mM Tris-HCl pH 7.4, 150 mM NaCl, 2.0 mM EDTA, 1% NP-40, 0.1% SDS and 0.5% sodium deoxycholate), supplemented with protease and phosphatase inhibitor cocktails (MilliporeSigma). Lysates were centrifuged at 10,000 g in refrigerated centrifuge set at 4 °C for 10 min. Supernatants were transferred to a new microfuge tube and protein concentration was measured using Pierce BCA protein assay kit (Thermo Fisher Scientific) according to manufacturer’s instructions. Lysates were mixed with appropriate volume of 3 × Laemmli buffer containing β-mercaptoethanol and boiled for 5 min. Protein samples were resolved on the appropriate % SDS-polyacrylamide gels and subjected to electrophoresis. After electrophoresis, proteins were transferred to nitrocellulose membrane for 1 h. Membranes were blocked in 5% blotting grade fat-free milk in TBS-T (Tris-Buffered Saline-0.1% Tween-20) for 1 h. After blocking, membranes were incubated with primary antibodies of interest for 1 h at room temperature or overnight at 4 °C. After primary antibody incubation, membranes were washed thrice in TBST, for 15 min each time. After washes, membranes were incubated in corresponding secondary antibodies at room temperature on rocker for 1 h, followed by 3 washes of TBST, 15 min each. After final wash, membranes were developed using ECL Western Blotting Substrate (Thermo Fisher Scientific) or infrared detection apparatus (LI-COR, Lincoln, NE).

### Lactate dehydrogenase activity

A549 and H460 cells were infected with lentiviruses encoding scramble or HOXC8 shRNAs. Post 4 days of infection, effect of HOXC8 depletion on LDH activity in the culture supernatants of cells was measured by Pierce™ LDH Cytotoxicity Assay Kit (#88953) (Thermo Fisher Scientific).

### Caspase-1 Activity

A549 and H460 cells were infected with lentiviruses encoding scramble or HOXC8 shRNAs. Post 4 days of infection, effect of HOXC8 depletion on Cellular Caspase-1 activity was determined by Caspase-1 Colorimetric Assay Kit (#K111) from BioVision as per supplier’s instructions.

### IL1β level measurement

The secretion of IL1β was analyzed using Interleukin-1β (human) ELISA Kit (Cayman, Cat. No. 583311). A549 cells or H460 cells were plated at 500,000 cells/well in 6 well plates overnight followed by lentiviral infection of scrambled control, HOXC8 shRNA1 or HOXC8 shRNA2 for 3 days. Cells were then washed and replenished with serum-free medium one day before supernatants and cell lysates were collected. Supernatants (100 µl/sample) were used for quantitation of IL1β secretion according to manufacturer’s protocol. Amount of cell lysates was determined and used for normalization of IL1β secretion.

### Immunoprecipitation

A549 or H460 cells were grown on 100-mm dish, washed twice with cold PBS and scraped gently on ice in lysis buffer (25 mM Tris-HCl pH 7.4, 150 mM NaCl, 1 mM EDTA, 1% NP-40 and 5% glycerol) containing freshly added protease and phosphatase inhibitors. Lysate transferred into the microfuge tube and placed on rotator for 1 h at 4 °C. After 1 h, lysate was centrifuged at 10,000 rpm for 5 min in a refrigerated centrifuge. Supernatant was transferred to a new microfuge tube and pre-cleared on rotator at 4 °C for 1 h with Dynabeads Protein G (Thermo Fisher Scientific) that were pre-washed with lysis buffer. Protein concentration in lysate was estimated by BCA method and equal amount of protein containing lysate was transferred to microfuge tubes containing either antibody of interest or a normal IgG, used as a negative control. These tubes were incubated on rotator for either 1 h at room temperature or overnight at 4 °C. After incubations, immunocomplexes were immobilized with Protein G Dynabeads at 4°C on rotator for 2 h. Immobilized immunocomplexes were washed thrice for 15 min each time on rotator with lysis buffer. After the last wash, lysis buffer was removed as much as possible and immunocomplexes were boiled with 3×Laemmli buffer containing beta-mercaptoethanol for 5 min. Immunocomplexes were resolved on SDS-PAGE and probed for proteins of interest by Western blotting.

### Immunofluorescence Staining

Cells were cultured on Lab-Tek chamber slides (Thermo Fisher Scientific) overnight. Next day, cells were fixed with warm 4% paraformaldehyde followed by three 5 min washes with PBS and then permeabilized with 0.2% Triton X-100 for 5 min. Following three PBS washes of 5 min each, cells were blocked with 5% BSA in PBS-T (PBS + 0.1% Tween 20) for 1 h. To detect ASC, slides were incubated with anti-ASC antibody overnight at 4 °C. Next day, cells were washed thrice with PBS-T for 5 min each and incubated with Alexa Fluor 488 dye conjugated goat anti-mouse antibody (Thermo Fisher Scientific) for 1 h at room temperature. To detect co-localization of HDAC1 and HOXC8, slides were co-incubated with mouse anti-HDAC1 mAb and rabbit anti-HOXC8 polyclonal for 2 h at room temperature followed by incubation with secondary antibodies (Alexa Fluor® 488 and Alexa Fluor® 568, Thermo Fisher Scientific) for another hour. All the subsequent procedure was performed in dark. After incubation with secondary antibodies, slides were washed three times with PBS-T for 5 min and any residual PBS was gently siphoned off. Slides were then mounted with ProLong Gold Antifade mountant with DAPI (Thermo Fisher Scientific) reagent and sealed with nail polish. Immunofluorescence staining was visualized under N-STORM coupled Nikon Eclipse Ti confocal microscope.

### Chromatin Immunoprecipitation (ChIP)

ChIP was performed to determine HOXC8, HDAC1 or RP-II occupancy at CASP1 promoter in A549 and H460 cells. Briefly, 25 million overnight grown cells per reaction tube were counted and cross-linked in final concentration of 1% formaldehyde for 10 min followed by addition of glycine to final concentration of 125 mM for 5 min to quench the cross-linking process. Cells were washed twice with cold PBS, centrifuged and pellet collected into 1.5 ml microfuge tube while the residual PBS aspirated out after brief centrifugation. Cells were suspended in ChIP lysis buffer (50 mM Tris-HCl pH 8.0, 150 mM NaCl, 1% SDS, 10 mM EDTA) supplemented with protease and phosphatase inhibitors and kept on ice for 10 min after which the suspension was gently re-suspended followed by sonication in Bioruptor Pico (Diagenode, Denville, NJ). Debris post sonication was cleared by centrifugation at 10,000 g for 5 min at 4 °C and supernatant transferred to microfuge tubes containing pre-washed Protein-G Dynabeads for preclearing on rotator for 1 h at 4 °C. Pre-cleared sheared chromatin was immunoprecipitated with antibody of interest or corresponding species-specific normal IgG overnight at 4 °C on rotator. Immunocomplexes were captured by incubation with Protein-G Dynabeads for 2 h at 4 °C on rotator. Captured immunocomplexes were washed sequentially with low salt (20 mM Tris-HCl pH 8.0, 100 mM NaCl, 0.1% SDS, 1% TritonX-100, 2 mM EDTA), high salt (20 mM Tris-HCl pH 8.0, 500 mM NaCl, 0.1% SDS, 1% TritonX-100, 2 mM EDTA) and LiCl buffer (25 mM LiCl, 1% NP-40, 1% sodium deoxycholate, 1 mM EDTA, 10 mM Tris-HCl pH8.0) for 15 min per wash at 4 °C. Immunocomplexes were eluted with 500 µL elution buffer (1%SDS in 0.1 M NaHCO3) on rotator at room temperature for 30 min followed by vortexing for 1 min. Formaldehyde cross-links of elute was reversed by adding 20 µL 5 M NaCl at 65 °C overnight. Protein component of the elute was digested by incubating elute at 45 °C with 40 µL 1 M Tris-HCl pH 7.0, 10 µL 0.5 M EDTA pH 8.0 and 1 µL of 20 mg/ml Proteinase K for 2 h. DNA in the elute was purified using standard phenol:chloroform extraction. CASP1 promoter sequence enrichment was determined by qPCR using primers amplifying region either near the transcription start site or spanning multiple HOX protein-binding consensus sequence TAATNN within CASP1 promoter. Sequences of the primers used are described in Supplementary Table S1.

### Promoter Assay

CASP1 promoter sequence of ~1450 bp was amplified using genomic DNA isolated from A549 cells. Primer sequences are provided in Supplementary Table S1. Purified promoter fragment was sub-cloned into the pGL3-basic vector (Promega, Madison, WI) that contains the firefly luciferase reporter gene. To determine CASP1 promoter activity, overnight-cultured scramble as well as HOXC8 depleted A549 and H460 cells were transfected with CASP1 promoter reporter plasmids using lipofectamine 3000. To normalize the difference in the transfection efficiencies, plasmid containing PGK promoter-driven Renilla luciferase gene (PGK-R/Luc, Promega) was transfected simultaneously as an internal control. Twenty four hours after transfection, cells were washed with PBS, lysed and luciferase activities were measured using the dual luciferase assay system (Promega) as per manufacturer’s instructions. The CASP1 promoter activity was calculated by dividing the firefly luciferase activity with Renilla luciferase activity.

### Quantitative RT-PCR (RT-qPCR)

Total cellular RNA was extracted using Trizol (ThermoFisher) followed by treatment with DNase I. Two micrograms of DNA-free RNA were reverse transcribed with ProtoScript® II First Strand cDNA Synthesis kit (New England Biolabs, Ipswich, MA). Generated cDNAs were then subjected to qPCR to measure the level of caspase-1 mRNA. The level of β-Actin mRNA was also measured and used for standardization. Sequences of the primers used are included in Supplementary Table S1.

### GST Pulldown Assay

Full length HDAC1 and various deletion constructs of HDAC1 were cloned into pGEXGP1. These constructs were transformed into BL21 competent cells. Corresponding GST-Fusion proteins were purified from bacterial lysates using Glutathione Sepharose® 4B beads. GST fusion protein bound beads were incubated with A549 cell lysate that was lysed in protease-phosphatase inhibitor cocktail supplemented lysis buffer consisting of 20 mM Tris-Cl pH 8.0, 200 mM NaCl, 1 mM EDTA, 0.5% NP-40 for 3 h at 4 °C. The beads were washed three times in lysis buffer. HOXC8 association was determined by western blotting.

### Tumorigenesis study

Athymic nude mice were obtained from Shanghai Laboratory Animal Center (Shanghai, China). A549 cells suspended in serum-free DMEM were mixed with Matrigel in 1:1 ratio and the mixture was injected into the flank of nude mice. After 3 weeks, a total of 1 OD Chol-Scrambled control or Chol-HOXC8 siRNA was injected at 3 different spots on each established tumor. Treatment was repeated once every 3 days and rate of tumor outgrowth was determined by measuring tumor volumes (V) which were calculated using formula of V = 0.5 x D_max_ x (D_min_)^2^, where D_max_ is the maximal tumor diameter and D_min_ is the corresponding perpendicular diameter. After 3 weeks of treatment, mice were euthanized and tumors were excised for weight measurement. Part of tumors were subjected to western blot analysis of HOXC8 and others were fixed for immunohistochemistry of HOXC8, caspase-1, Ki67, and cleaved caspase-3. All procedures were conducted with animal welfare considerations and approved by the Ethical Committee of Shanghai University of Traditional Chinese Medicine.

### Bioinformatics and statistical analyses

HOXC8 expression in pan-cancer was analyzed with the aid of eBioportal using Dataset from “Pan-cancer analysis of whole genomes” project. To compare the level of HOXC8 expression between normal and NSCLC tissues, HOXC8, HDAC1 and HDAC2 expression data (LUAD, *n* = 576; LUSC, *n* = 553) was downloaded from Xena (http://xena.ucsc.edu/). Plots for HOXC8, HDAC1, and HDAC2 expression in normal *vs* tumor were generated in GraphPad Prism with whiskers down to the minimum value and up to the maximum value in each group. To analyze the correlation between CASP1 and HOXC8, HDAC1 or HDAC2 expression, CASP1 expression data were also downloaded from Xena. Pearson’s r and two-tailed *P*-value were performed using GraphPad Prism. Kaplan–Meier curves were generated using http://kmplot.com. All experiments were performed in triplicates. Results of each experiment were reported as the means of experimental replicates. All statistical analyses were performed using PRISM 8.3 with unpaired *t* test (two-tailed). Error bars represent the standard error of means (SEM). For all tests, *P* < 0.05 was considered statistically significant.

## Results

### HOXC8 is overexpressed in lung cancer and is required for the survival of lung cancer cells

**Fig. 1:**
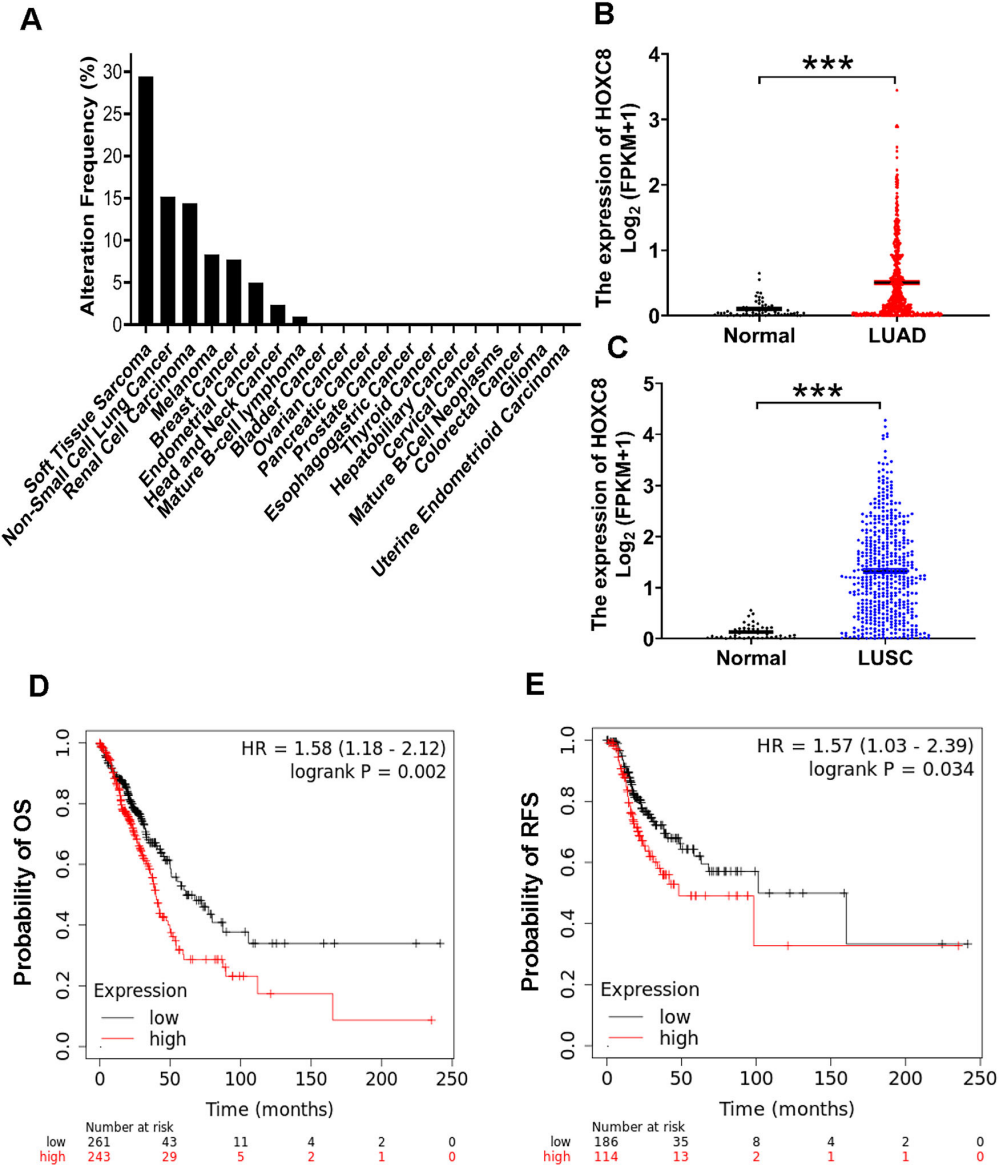
HOXC8 expression is preferentially higher in LUAD and negatively associated with patient survival. **A** Frequency of high HOXC8 expression in various cancer types. **B** Level of HOXC8 mRNA in normal lung and LUAD tissue. ***P < 0.001. **C** Level of HOXC8 mRNA in normal lung and LUSC tissue. ***P < 0.001. **D** Kaplan–Meier overall survival plot of HOXC8. **E** Kaplan–Meier recurrence-free survival plot of HOXC8.

Dysregulated HOXC8 expression has been reported in a variety of cancer types. To have a systemic view of HOXC8 expression in various cancer types, we utilized eBioPortal to compare the level of HOXC8 mRNA across 30 cancer categories using dataset from Pan-cancer analysis of whole genomes [16] and found that the level of HOXC8 mRNA was high in 8 cancer categories (Fig. 1A). We paid specific attention on non-small cell lung cancer (NSCLC) because more people in the United States die from lung cancer than any other type of cancer and NSCLC account for about 80–85% of all lung cancers [17]. Analysis of The Cancer Genome Atlas (TCGA) lung cancer datasets revealed that HOXC8 expression was significantly higher in NSCLC that included both lung adenocarcinoma (LUAD) and lung squamous cell carcinoma (LUSC), compared with corresponding normal tissue (Fig. 1B, C). Additionally, higher expression of HOXC8 is inversely associated with both the overall survival (OS) and recurrence free survival (RFS) of patients with LUAD (Fig. 1D, E). However, such statistically significant associations were not observed in LUSC (Supplementary Fig. S1A and S1B). Together, these analyses suggest that dysregulated expression of HOXC8 may play a prominent role in the tumorigenesis of NSCLC, particularly LUAD.

**Fig. 2:**
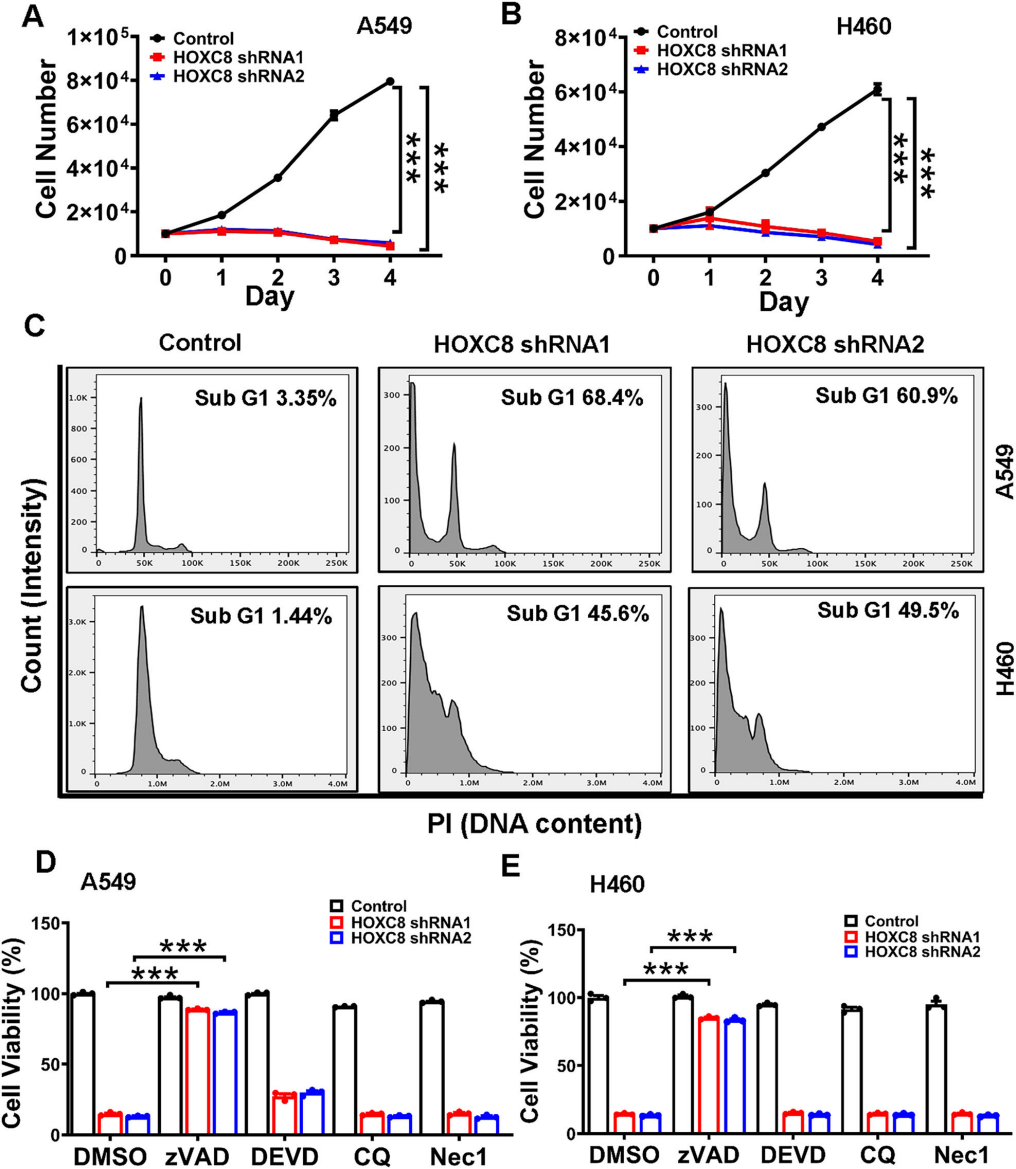
Knockdown of HOXC8 leads to caspase-dependent non-apoptotic cell death of LUAD cells. A549 (**A**) and H460 cells (**B**) were lentivirally transduced with either Scrambled sequence (control) or HOXC8 shRNAs for 2 days followed by counting cells using Cellometer. Data are means ± SEM. ***P < 0.001. **C** A549 and H460 cells were lentivirally transduced with Scrambled sequence (control) or HOXC8 shRNAs for 3 days followed by PI-based flow cytometry to determine cell cycle progression. A549 (**D**) and H460 cells (**E**) were infected with lentiviral vector containing Scrambled sequence (control) or HOXC8 shRNAs for 2 days and then cultured in the presence of vehicle (DMSO), 10 µM zVAD, 10 µM DEVD, 50 µM chloroquine (CQ) or 10 µM necrostatin for 2 days followed by an MTT assay to analyze cell viability. Data are means ± SEM. ***P < 0.001.

To determine whether HOXC8 is critically involved in the growth/survival of LUAD cells, we endeavored to a complete knockout of HOXC8 in LUAD cell lines A549 and H460 through CRISPR/CAS9-based sgRNA approach without success, indicating that HOXC8 may be essential for the survival of these two cell lines. We next lentivirally introduced HOXC8 shRNAs into A549 and H460 cells. Remarkably, knockdown of HOXC8 in these cells led to a very noticeable reduction in cell growth as early as 24 h post-transduction compared to the cells infected with scrambled shRNA lentivirus (Fig. 2A, B). Day 3 and 4 of post-transduction brought an apparent cell death effect based on cell counting (Fig. 2A, B) and as visualized by profound increase in floating cells (Fig. S2). In contrast, A549 and H460 cells transduced with Scrambled shRNA maintained cell growth trajectory (Fig. 2A, B). To further experimentally corroborate these observations, we performed flow cytometry analysis of cell cycle with propidium iodide on HOXC8-knockdown A549 and H460 cells along with their respective Scrambled control. In line with the cell counting and visualization, HOXC8-depleted cells exhibited a dramatic increase in the sub-G1 population (A549 cells: 68.4 and 60.9% for HOXC8 shRNA1 and shRNA2, respectively, compared to 3.35% in Scrambled control; H460 cells: 45.6 and 49.5% for HOXC8 shRNA1 and shRNA2, respectively, compared with 1.44% in Scrambled control) (Fig. 2C). These results, consistent with the failure to generate HOXC8 knockout cells, further support that HOXC8 is essential for LUAD cell survival.

Appearance of sub-G1 population (diminished DNA content) often results from DNA damage/fragmentation, the hallmark of apoptosis. We thus investigated whether apoptosis contributed to the cell death led by HOXC8 knockdown. To our surprise, western blot analysis failed to reveal caspase-3 cleavage in A549 and H460 cells with HOXC8 knockdown (Fig. S3). In a parallel experiment, we cultured HOXC8-knockdown cells in the presence of DEVD (caspase-3 inhibitor), zVAD (pan-caspase inhibitor), chloroquine (autophagy inhibitor) or necrostatin-1 (necroptosis inhibitor). As would-be expected, chloroquine and necrostatin-1 did not prevent cell death induced by HOXC8 knockdown (Fig. 2D, E), as neither autophagy nor necroptosis accompanies with DNA damage/fragmentation. Although DEVD exhibited little protective effect on the survival of HOXC8-knockdown A549 cells, zVAD almost completely blocked cell death induced by HOXC8 knockdown in both A549 and H460 cells (Fig. 2D, E). These results suggest that cell death led by HOXC8 knockdown is caspase-dependent but independent of apoptosis-associated caspase-3.

### HOXC8 knockdown triggers cell death in a caspase-1 dependent manner

**Fig. 3:**
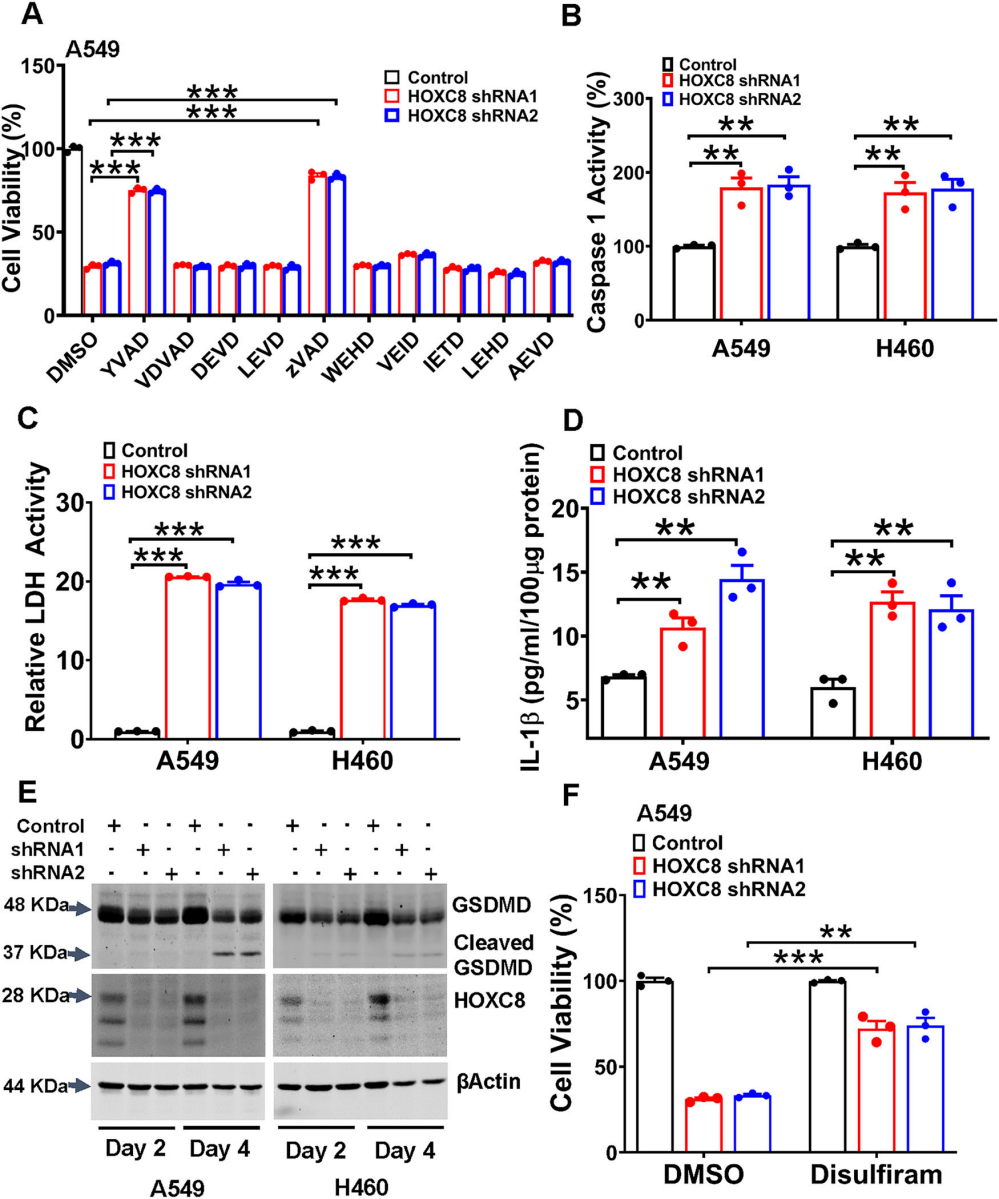
Knockdown of HOXC8 induces caspase-1-mediated pyroptosis in LUAD cells. **A** A549 cells were infected with lentiviral vector containing Scrambled sequence (control) or HOXC8 shRNAs for 2 days and then cultured in the presence of vehicle (DMSO) or 10 µM inhibitor of each individual caspase for 2 days followed by an MTT assay to analyze cell viability. Data are means ± SEM. ***P < 0.001. **B** A549 and H460 cells were lentivirally transduced with either Scrambled sequence (control) or HOXC8 shRNAs for 3 days followed by analysis of caspase-1 activity. Data are means ± SEM. **P < 0.01. **C** A549 and H460 cells were lentivirally transduced with either Scrambled sequence (control) or HOXC8 shRNAs for 3 days and conditioned media were then collected to measure LDH activity. Data are means ± SEM. *n* = 3. ***P < 0.001. **D** A549 and H460 cells were lentivirally transduced with either Scrambled sequence (control) or HOXC8 shRNAs for 3 days and conditioned media were then collected to measure the level of IL-1β. Data are means ± SEM. **P < 0.01. **E** A549 and H460 cells were lentivirally transduced with either Scrambled sequence (control) or HOXC8 shRNAs for 2 or 4 days followed by western blotting to detect gasdermin D (GSDMD) and βActin with the respective antibodies. **F** A549 cells were lentivirally transduced with either Scrambled sequence (control) or HOXC8 shRNAs for 2 days and then culture in the presence of vehicle (DMSO) or 10 µM disulfiram for another 2 days, followed by an MTT assay to analyze cell viability. Data are means ± SEM. **P < 0.01; ***P < 0.001.

To characterize the specific caspase mediating cell death induced by HOXC8 knockdown, we cultured HOXC8-knockdown cells in the presence of inhibitors for each individual caspases – namely YVAD for caspase-1, VDVAD for caspase-2, DEVD for caspase-3/7, LEVD for caspase-4, WEHD for caspase-5, VEID for caspase-6, IETD for caspase-8, LEHD for caspase-9, AEVD for caspase-10 and zVAD for pan-caspase. MTT assay showed that only YVAD and zVAD were able to prevent cell death induced by HOXC8 knockdown in both A549 and H460 cells (Fig. 3A and S4). To further confirm the involvement of caspase-1, we measured caspase-1 activity in A549 and H460 cells transduced with Scrambled or HOXC8 shRNA and found that caspase-1 activity was elevated by over 75% in HOXC8 knockdown cells compared to their respective Scrambled controls (Fig. 3B).

Caspase-1 is the mediator of canonical inflammasome-dependent pyroptosis [9]. To determine whether HOXC8-knockdown cells underwent cell death through pyroptosis, we compared released lactate dehydrogenase (LDH) between Scrambled control and HOXC8 shRNA-transduced cells. Strikingly, we found that LDH activity was 18 to 21-fold higher in HOXC8 knockdown cells compared to their respective controls (Fig. 3C). Consistent with the fact that IL1β is a substrate of caspase-1, there was more than a fold of increase in IL1β secretion in HOXC8-knockdown cells over the control (Fig. 3D). Also in line with GSDMD being a caspase-1 substrate, HOXC8 knockdown led to a reduction of pro-GSDMD and the appearance of cleaved GSDMD in both A549 and H460 cells (Fig. 3E). To further confirm the essence of GSDMD pore formation in cell death, cells were treated with Disulfiram, a GSDMD pore formation blocker [18]. MTT assay showed that Disulfiram blocked more than half of the cell death observed in HOXC8-knockdown cells (Fig. 3F, S5 and S6). Collectively, these results strongly suggest that HOXC8-knockdown LUAD cells undergo cell death through caspase-1-dependent pyroptosis.

### Knockdown of HOXC8 markedly increases caspase-1 expression without the participation of ASC

**Fig. 4:**
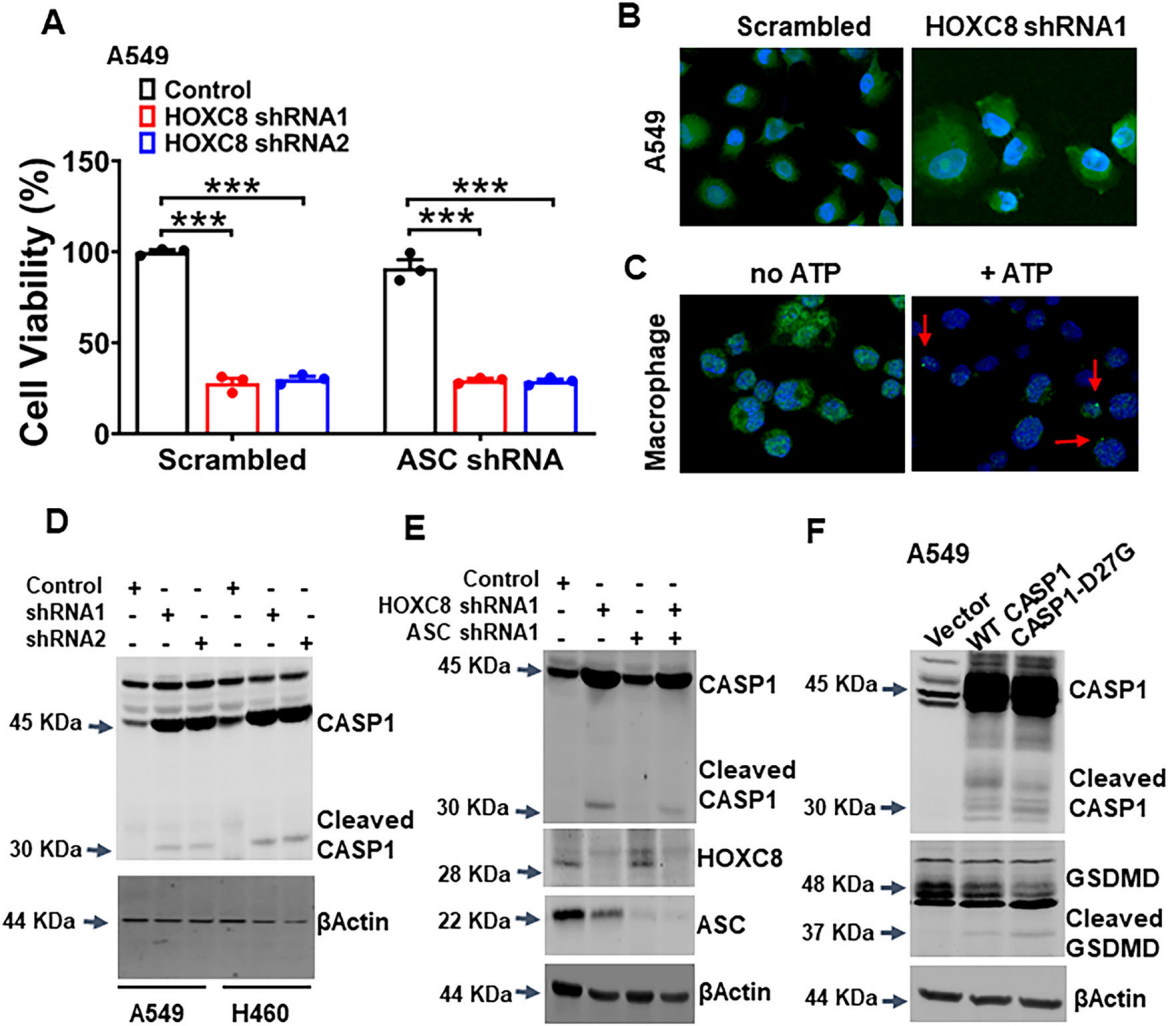
Pyroptosis induced by knockdown of HOXC8 occurs through the augmentation of caspase-1 abundance. **A** A549 cells were first lentivirally transduced with Scrambled sequence or ASC shRNA for 2 days and then further transduced with either Scrambled sequence (control) or HOXC8 shRNAs for 3 days, followed by an MTT assay. Data are means ± SEM. ***P < 0.001. **B** A549 cells were lentivirally transduced with either Scrambled sequence (control) or HOXC8 shRNA1 for 3 days and then fixed for immunofluorescence staining with ASC antibody. DAPI was included for nuclear staining. **C** Murine macrophages were treated with ATP for 10 min and then fixed for immunofluorescence staining with ASC antibody. DAPI was included for nuclear staining. **D** A549 and H460 cells were lentivirally transduced with either Scrambled sequence (control) or HOXC8 shRNAs for 4 days and then lysed for western blotting to detect caspase-1 (CASP1) and βActin with their respective antibodies. **E** A549 cells were first lentivirally transduced with scramble or ASC shRNA for 2 days and then further transduced treated with either Scrambled sequence (control) or HOXC8 shRNA1 for 3 days, and then lysed for western blotting to detect caspase-1 (CASP1), HOXC8, ASC and βActin with the respective antibodies. **F** A549 cells were transfected with 2 µg of plasmid encoding wild-type caspase-1 or caspase-1/D27G mutant for 24 h and then lysed for western blotting to detect caspase-1 (Casp1), GSDMD and βActin with the respective antibodies.

Canonical activation of caspase-1 occurs through ASC-facilitated oligomerization on the inflammasome in immune cells [19] although caspase-1 can also be activated through NLRP1b in the absence of ASC [20]. Since ASC was readily detected in A549 cells, we lentivirally introduced ASC shRNA into A549 cells to silence ASC expression (Fig. S7). Surprisingly, MTT assay showed that depleting ASC was unable to prevent cell death caused by HOXC8-knockdown (Fig. 4A and S8). Immunofluorescence staining with anti-ASC antibody failed to reveal obvious ASC speck in HOXC8-knockdown cells (Fig. 4B), while ASC specks were readily seen in murine macrophages stimulated with ATP (Fig. 4C). These results suggest that ASC is not involved in pyroptosis triggered by HOXC8 silencing.

We recently discovered that caspase-1 can be activated through its excessive accumulation without the involvement of ASC or the inflammasome subunit NLRP3 [14]. To determine whether a similar mechanism mediated HOXC8 depletion-triggered pyroptosis, we performed western blot to examine the level of caspase-1 in scrambled control and HOXC8-knockdown A549 and H460 cells. The abundance of caspase-1 was greatly elevated in cells with HOXC8 knockdown compared to the control (Fig. 4D). In addition, the emergence of cleaved caspase-1 (active caspase-1) was seen in cells transduced with HOXC8 shRNAs (Fig. 4D), ratifying the activation of caspase-1 in these cells. Subsequently, we first silenced ASC in A549 cells followed by depleting HOXC8. Knockdown of ASC neither altered the endogenous level of caspase-1 nor prevented HOXC8 depletion-led increase in caspase-1 abundance and subsequent cleavage (Fig. 4E). To further confirm that excessive caspase-1 could lead to caspase-1 activation without ASC, we overexpressed wild-type caspase-1 or caspase-1/D27G mutant, which is unable to interact with ASC, in A549 cells. Western blot analysis revealed the appearance of cleaved caspase-1 in both wild-type and mutant caspase-1 transfected cells (Fig. 4F). Moreover, a similar pattern of cleaved GSDMD was also detected in cells ectopically expressing either wild-type or mutant caspase-1 (Fig. 4F). These results suggest that, in HOXC8-knockdown cells, caspase-1 is activated through augmented caspase-1 abundance without the involvement of ASC.

### HOXC8 epigenetically controls caspase-1 expression in lung cancer cells

**Fig. 5:**
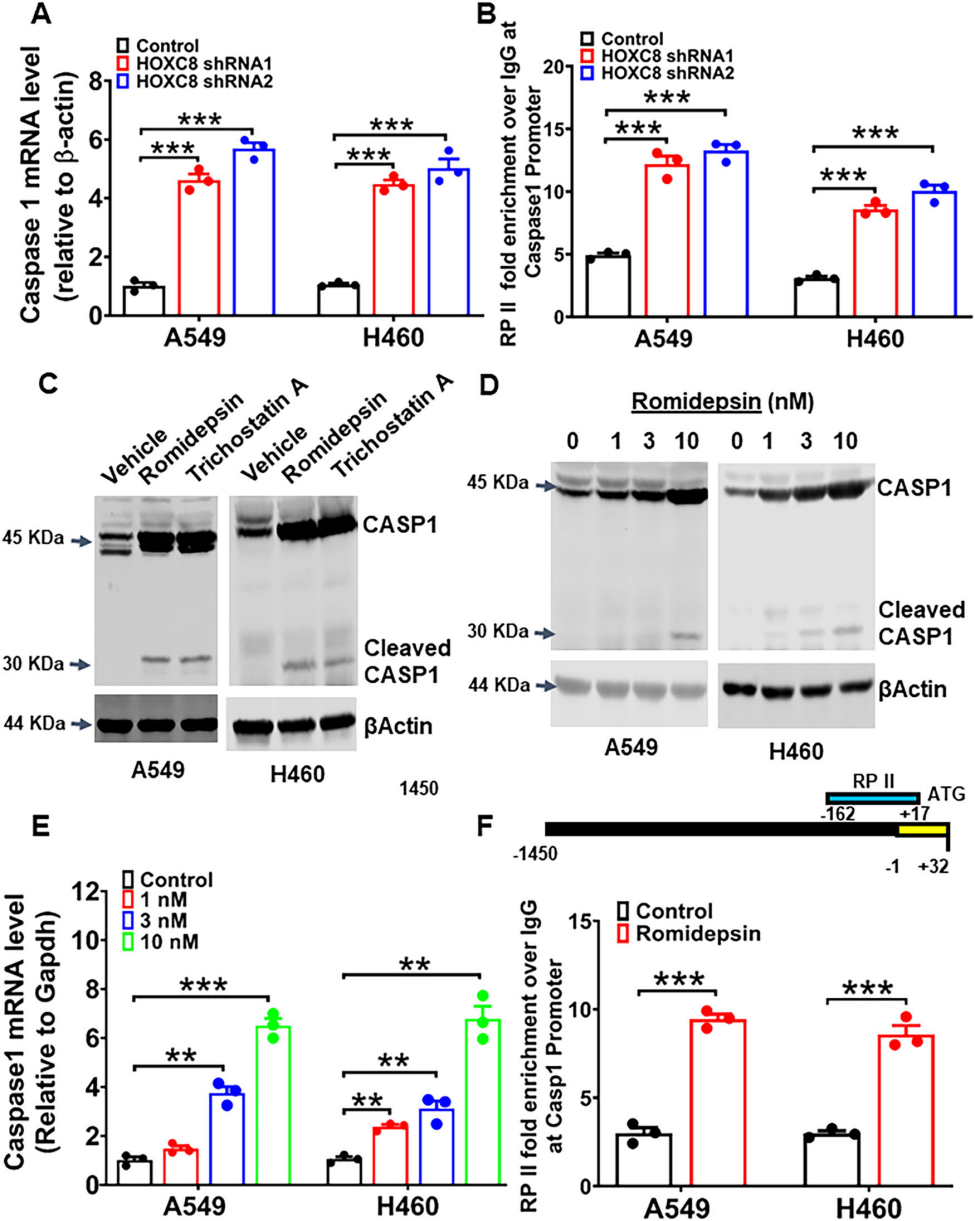
HOXC8 epigenetically controls caspase-1 expression in LUAD cells. **A** A549 and H460 cells were lentivirally transduced with either Scrambled sequence (control) or HOXC8 shRNAs for 3 days and then extracted for total RNA to analyze the level of caspase-1 mRNA by RT-qPCR. Level of βActin mRNA was used for standardization. Data are means ± SEM. ***P < 0.001. **B** A549 and H460 cells were lentivirally transduced with either Scrambled sequence (control) or HOXC8 shRNAs for 3 days and then subjected to RP-II ChIP followed by qPCR to analyze RP-II occupancy at *CASP1* promoter. Data are means ± SEM. ***P < 0.001. **C** A549 and H460 cells were treated with 100 nM Trichostatin A (TSA) or 10 nM Romidepsin for 2 days and then lysed for western blotting to detect caspase-1 (CASP1) and βActin with their respective antibodies. **D** A549 and H460 cells were treated with varying concentration of Romidepsin for 2 days and then lysed for western blotting to detect caspase-1 (CASP1) and βActin with their respective antibodies. **E** A549 and H460 cells were treated with varying concentration of Romidepsin for 1 day and then extracted for total RNA to measure caspase-1 mRNA by RT-qPCR. Level of βActin mRNA was used for standardization. Data are means ± SEM. **P < 0.01. ***P < 0.001. **F** A549 and H460 cells were treated with 10 nM Romidepsin for 2 days and then subjected to RP-II ChIP followed by qPCR to analyze RP-II occupancy at *CASP1* promoter. Data are means ± SEM. ***P < 0.001.

To define the mechanism associated with elevated caspase-1 abundance in HOXC8-knockdown cells, we assessed the level of caspase-1 mRNA between control and cells with HOXC8 knockdown by qRT-PCR. The level of caspase-1 mRNA was more than 4-fold higher in HOXC8-knockdown cells compared to cells transduced with Scrambled control (Fig. 5A). Further qPCR-based chromatin immunoprecipitation (ChIP) with RNA polymerase II (RP-II) antibody showed that the knockdown of HOXC8 led to approximately 3-fold increase in RP-II occupancy at the *CASP1* promoter (Fig. 5B), suggesting that enhanced caspase-1 expression in HOXC8-depleted cells is caused by accelerated *CASP1* transcription. Astonishingly, a reporter gene plasmid containing 1450 bp *CASP1* gene promoter sequence exhibited almost identical luciferase activity between scrambled control and HOXC8 knockdown cells (Fig. S9). These results indicate that HOXC8 may regulate *CASP1* transcription through the chromosome accessibility around the *CASP1* gene rather than regulating the expression of particular transcription factor(s).

Chromosome accessibility may be controlled by histone acetylation. To test whether the histone acetylation controls HOXC8 regulation of *CASP1* gene accessibility, we first investigated the relevance of HDACs in caspase-1 expression by treating A549 and H460 cells with Trichostatin A (TSA), a potent inhibitor of class I/II HDACs, and Romidepsin, a specific inhibitor of class I HDACs. Western blot analysis showed that both inhibitors effectively increased the abundance of caspase-1 and led to caspase-1 activation (appearance of cleaved caspase-1) (Fig. 5C). To ensure the specificity of HDAC inhibitor, we treated cells with increasing concentrations of Romidepsin and found that Romidepsin dose-dependently elevated the level of caspase-1 (Fig. 5D). Notably, the presence of cleaved caspase-1 was apparent in cells treated with 10 nM Romidepsin (Fig. 5D), consistent with the notion that excessive caspase-1 accumulation is sufficient to cause caspase-1 activation.

We next examined the effect of Romidepsin on the level of caspase-1 mRNA in A549 and H460 cells. Similar to what we observed with caspase-1 protein, Romidepsin dose-dependently enhanced the level of caspase-1 mRNA (Fig. 5E). Moreover, ChIP with anti-RP-II antibody showed that Romidepsin treatment resulted in approximately 3-fold increase in RP-II occupancy in *CASP1* promoter over the vehicle-treated control cells (Fig. 5F). Taken together, these results suggest that class I HDACs are involved in *CASP1* transcription by controlling chromosome accessibility around *CASP1* promoter.

### HOXC8 recruits HDAC1 to *CASP1* promoter to suppress caspase-1 expression

**Fig. 6:**
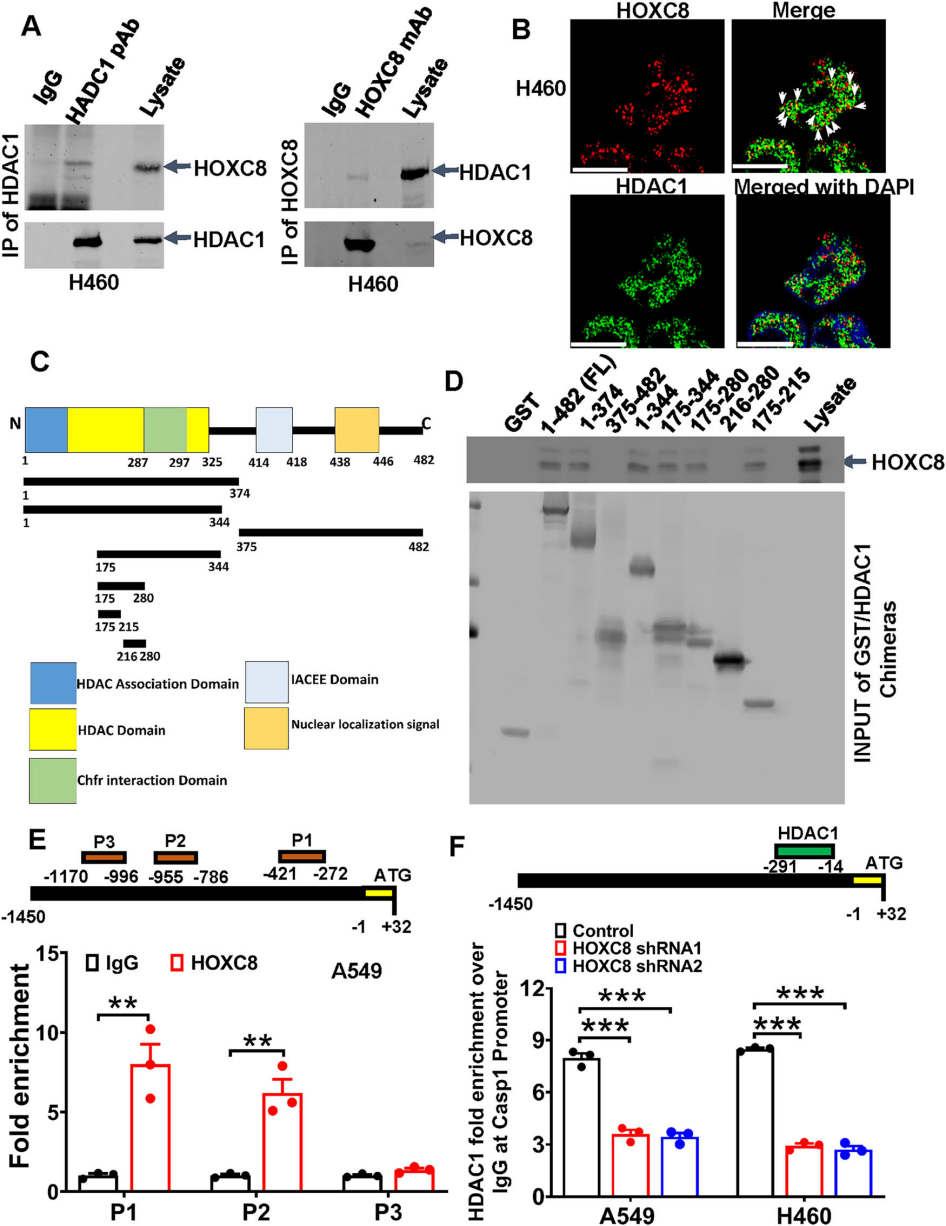
HOXC8 recruits HDAC1 to *CASP1* promoter to suppress caspase-1 expression. **A** H460 cells were immunoprecipitated with either HDAC1 or HOXC8 antibody and the immunoprecipitates were subjected to western blot to detect HOXC8 or HDAC1, respectively. **B** H460 cells were subjected to immunofluorescence staining to detect HOXC8 and HDAC1. Data are the representative of six independent experiments. **C** Schematic diagram of GST-tagged HDAC1 deletion constructs. **D** A549 cell lysates were incubated with GST beads containing various HDAC1 fragments on ice for 2 h. After 4 washes, beads were boiled and then subjected to western blot analysis to detect HOXC8. **E** A549 cells were subjected to ChIP with IgG or rabbit anti-HOXC8 polyclonal antibody followed by qPCR to analyze the occupancy of HOXC8 in P1, P2, and P3 regions of *CASP1* promoter. Data are means ± SEM. **P < 0.01. **F** A549 and H460 cells were lentivirally transduced with either Scrambled sequence (control) or HOXC8 shRNAs for 3 days and then subjected to HDAC1 ChIP followed by qPCR to analyze HDAC1 occupancy in the indicated region of *CASP1* promoters. Data are means ± SEM. ***P < 0.001.

The findings that both HOXC8 and class I HDAC(s) negatively regulated *CASP1* transcription prompted us to hypothesize that HOXC8 recruited class I HDAC(s) to *CASP1* gene to suppress its transcription. To test this hypothesis, we initially performed co-immunoprecipitation experiments in both A549 and H460 cells using either antibody against HDAC1 or HOXC8. Western blot analysis showed that HOXC8 was present in the HDAC1 immunoprecipitates, while HDAC1 was in the HOXC8 immunoprecipitates (Fig. 6A and S10A). Subsequent confocal microscope-based immunofluorescence staining also displayed the co-localization of HOXC8 and HDAC1 in both A549 and H460 cells and, as expected, the interaction appeared restricted to the nucleus (Fig. 6B and S10B). To map the region in HDAC1 required for its interaction with HOXC8, we generated GST fusion proteins spanning different regions of HDAC1 (Fig. 6C). GST pulldown assay showed that region of HDAC1 interacting with HOXC8 was from amino acid residues 175 to 215 (Fig. 6D), which is within the HDAC domain.

To functionally link HOXC8 and HDAC1 to suppressed *CASP1* gene transcription, we performed ChIP using anti-HOXC8 antibody in A549 and H460 cells. Q-PCR with specific primer sets for various *CASP1* promoter regions revealed that occupancy of HOXC8 mainly occurred regions near the nucleotides −421 to −272 and −955 to −786 of the *CASP1* promoter, in which multiple TAAT, the putative HOX binding [21] could be identified (Fig. 6E, Figs. S11 and S12). ChIP with anti-HDAC1 antibody also exhibited robust HDAC1 occupancy around the *CASP1* promoter (Fig. 6F). However, knockdown of HOXC8 led to 55-65% reduction in HDAC1 occupancy at the *CASP1* promoter (Fig. 6F). These results are consistent with our hypothesis that HOXC8 recruits HDAC1 to the *CASP1* promoter to suppress caspase-1 expression in LUAD cells. Moreover, our hypothesis is also consistent with the fact that HOXC8 and caspase-1 expression were inversely associated in LUAD (Pearson’s *r* = −0.27 and *P* < 0.0001) (Fig. S13).

### Lipid-conjugated HOXC8 siRNA deters tumor development of LUAD cells

**Fig. 7:**
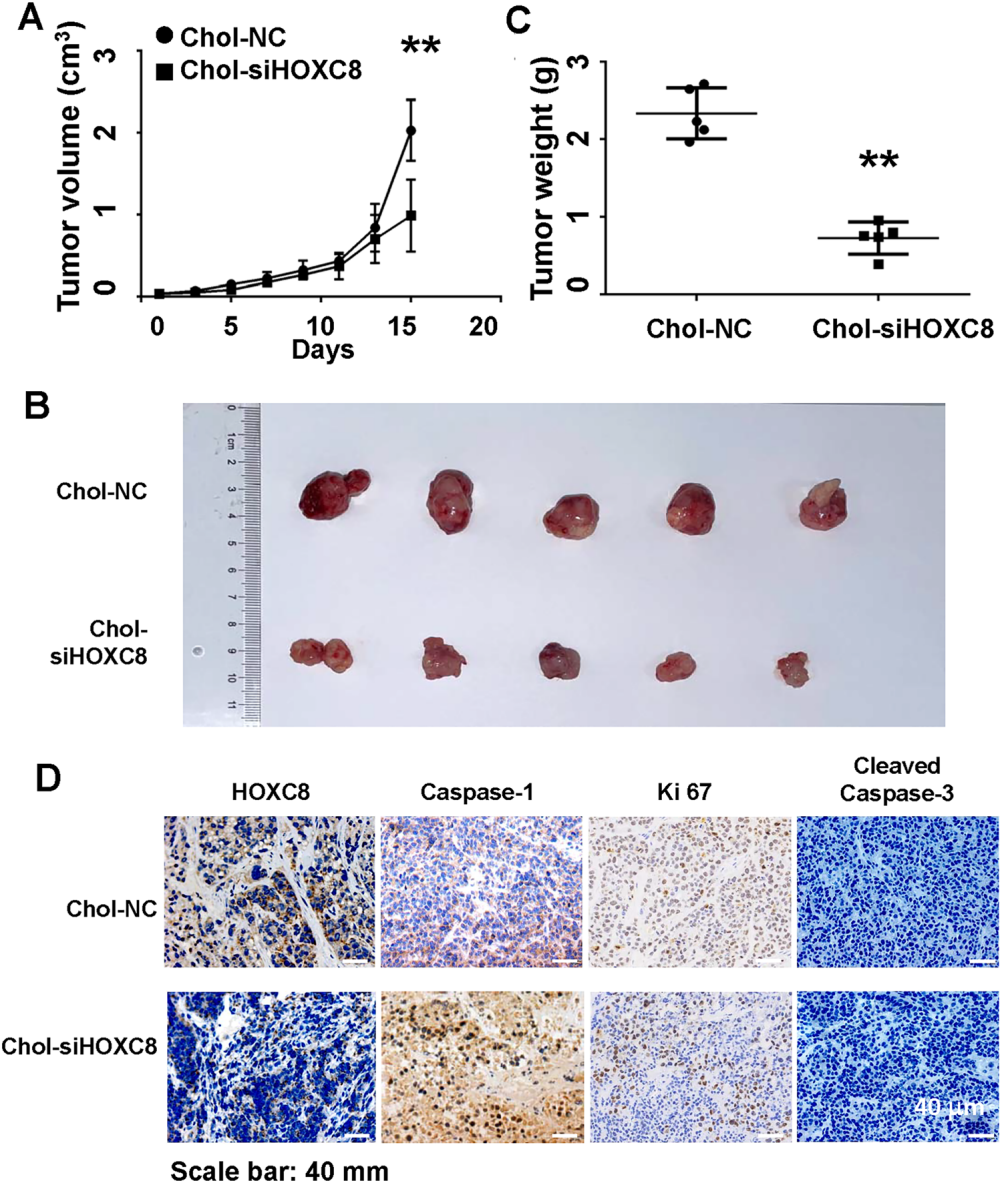
Cholesterol-conjugated HOXC8 siRNA suppresses tumor growth of A549 cells. **A** A549 cells (10^6^ cells/mouse) in Matrigel (mixed in 1:1 ratio) were injected to athymic nude mice for 3 week followed by administering 1 OD Chol-Scrambled sequence (Chol-NC) or Chol-HOXC8 siRNA (Chol-siHOXC8) directly on established tumors once every three days for 3 weeks. Rate of tumor development was determined by measuring tumor volume once every three days. Data are means ± SEM (*n* = 5). **P < 0.01 (Chol-siHOXC8 *vs* Chol-NC). **B** Image of tumors excised form tumor-bearing mice treated with Chol-NC or Chol-siHOXC8. **C** Weight of tumors excised from tumor-bearing mice treated with Chol-NC) or Chol-siHOXC8). Data are means ± SEM. **P < 0.01. **D** Representative pictures of IHC staining on HOXC8, caspase-1, Ki67 and cleaved caspase-3 in tumor tissues.

The ability of HOXC8 shRNAs to potently induce pyroptosis led us to investigate their effect on tumor development of LUAD cells. A549 cells were subcutaneously injected into athymic nude mice at left flank for 3 week followed by directly injecting cholesterol-conjugated control (Chol-NC) or HOXC8 siRNA (Chol-siHOXC8) to established tumors once every other day for 2 weeks. As indicated by tumor volumes, tumors developed slower in mice receiving intratumoral injection of Chol-siHOXC8 than those in mice receiving Chol-NC (Fig. 7A). At the end of treatment, tumors were excised from euthanized animals and weighed. Tumors recovered from Chol-siHOXC8 group was much smaller than those from Chol-NC group (Fig. 7B, C). These results suggest that lipid-conjugated HOXC8 siRNA is capable of suppressing tumor development of LUAD cells. To ensure that deterred tumor development was linked to pyroptosis led by the reduction of HOXC8, we performed immunohistochemistry to analyze the abundance of HOXC8, caspase-1, Ki67 and cleaved caspase-3 in tumors excised from Chol-NC and Chol-siHOXC8 groups. Staining of HOXC8 was much lower in tumors of Chol-siHOXC8 group compared to those of Chol-NC group (Fig. 7D and Fig. S14A), confirming the effectiveness of HOXC8 depletion. There was little caspase-1 but strong Ki67 staining in tumors excised from Chol-NC group (Fig. 7D and Fig. S14B and S14C). In contrast, tumors derived from Chol-siHOXC8 group displayed strong caspase-1 but greatly reduced Ki67 staining (Fig. 7D and Fig. S14B, C). Additionally, we were unable to detect cleaved caspase-3 staining in tumors derived from both groups (Fig. 7D). These results provide further evidence that HOXC8 knockdown suppress tumor development through caspase-1-associated pytoptosis rather than apoptosis.

To determine whether deterred tumorigenesis in Chol-siHOXC8 group was accompanied with altered levels of HDAC1 or HDAC2, we compared the intensities of HDAC1 and HDAC2 staining between tumors excised from Chol-NC and Chol-siHOXC8 groups. Immunohistochemistry staining with respective antibodies failed to reveal apparent difference in HDAC1 or HDAC2 intensity between Chol-NC and Chol-siHOXC8 groups (Fig. S15). Further analysis of TCGA datasets showed that both HDAC1 and HDAC2 expression were higher in LUAD compared to normal tissue (Fig. S16A). However, the expression of HOXC8 was not greatly correlated with either HDAC1 or HDAC2 (Fig. S16B). Collectively, these results suggest HOXC8 is not involved in HDAC1 and HDAC2 expression in LUAD.

## Discussion

We previously revealed that the ratio of miR-196 to HOXC8 mRNA is correlated with metastatic behaviors in breast cancer [15]. Later studies showed that HOXC8 is involved in breast cancer progression by either transcriptionally upregulating cadherin-11 expression [4, 22] or acting as a transcriptional repressor of the tumor suppressor Embigin [5]. Although the underlying mechanisms were not clearly demonstrated, HOXC8 has also been reported to be associated with glioma [1], pancreatic duct carcinoma [6], and prostate cancer [2]. Using the web-based eBioPortal and Xena, we found that HOXC8 is highly expressed in NSCLC (both LUAD and LUSC), and high HOXC8 expression is correlated with poorer survival in LUAD patients. To delve the potential role of HOXC8 in LUAD, we found that loss of HOXC8 resulted in caspase-1/GSDMD-mediated pyroptosis. Surprisingly, ASC/inflammasome was dispensable for pyroptosis occurring in HOXC8-depleted cells. Instead, we observed that knockdown of HOXC8 epigenetically increased caspase-1 expression. Mechanistically, we showed that HOXC8 repressed caspase-1 expression by recruiting HDAC1 to the *CASP1* gene promoter in LUAD cells.

Caspase-1 is activated in a two-step mechanism including proximity-induced oligomerization and autoprocessing within the catalytic domain [23]. Since caspase-1 exists as a monomer and multimers can only form when monomers are present at a sufficiently high concentration [24], which explains why caspase-1 is typically recruited to the ASC/inflammasome for activation. We initially hypothesized that an ASC/inflammasome-dependent mechanism was responsible for pyroptosis occurring in HOXC8-depleted cells. To our surprise, we found that knockdown of ASC displayed little effect on HOXC8 knockdown-led cell death or caspase-1 activation. Moreover, we did not observe ASC specks in HOXC8-depleted cells. These observations indicate that an ASC/inflammasome-independent mechanism facilitates pyroptosis in LUAD cells.

Caspase-1 is monomeric when free in the cytosol because its dimer dissociation constant is much greater than its endogenous intracellular concentration (e.g., Kd ∼ 110 μM *vs* 100 nM concentration in THP1 cells) [25]. We reasoned that caspase-1 dimerization and subsequent activation could occur without inflammasome if its intracellular concentration is sufficiently high. This reasoning is supported by our recent finding that DHA induces caspase-1 activation through translational augmentation of caspase-1 protein [14]. In this study, we detected greatly increased level of caspase-1 protein in HOXC8-depleted cells. To further support this reasoning, we found that overexpressing either wild-type caspase-1 or caspase-1 mutant incapable of interacting with ASC/inflammasome was able to undergo caspse-1 activation and pyroptosis. Collectively, these results suggest that HOXC8 knockdown induces caspase-1 activation and subsequently pyroptosis through the substantial accumulation of caspase-1 protein in LUAD cells.

In an effort to elucidate molecular mechanism underlying caspase-1 protein accumulation, we revealed that knockdown of HOXC8 increased the level of caspase-1 mRNA and the occupancy of RP-II in the *CASP1* promoter while not altering the activity of *CASP1* promoter reporter plasmid, suggesting that HOXC8 represses *CASP1* transcription through a histone-involved epigenetic mechanism in lung cancer cells. In fact, class I HDAC inhibitors including TSA and Romidepsin massively increased the abundance of caspase-1 while HOXC8 and HDAC1 were in the same complex. HDAC1 catalyzes the deacetylation of lysine residues on the N-terminal part of the core histones and histone deacetylation, resulting in epigenetic repression [26]. The specificity of HDAC1 is controlled by the component(s) of multi-protein HDAC1 complex [27, 28]. For example, ICBP90, an E2F-1 target, recruits HDAC1 to promoter regions of various tumor suppressor genes, thus promoting tumor cell proliferation [29]. In this study, we found that both HOXC8 and HDAC1 are bound to the *CASP1* promoter but removal of HOXC8 abolished HDAC1 binding to the *CASP1* promoter. Taken together, we conclude that HOXC8 suppresses caspase-1 expression by recruiting HDAC1 to the *CASP1* promoter in LUAD cells.

The ability of HOXC8 knockdown to trigger pyroptosis in lung cancer cells indicates that delivery of HOXC8 siRNA may possess tumor-inhibitory capability. With limited number of athymic nude mice, we showed that lipid-conjugated HOXC8 siRNA deterred tumor development. IHC of tumor sections from mice receiving HOXC8 siRNA displayed strong caspase-1 but reduced Ki67 staining without alteration of cleaved caspase-3 levels, indicating that tumor suppression was due to pyroptosis. Our finding that depleting HOXC8 triggers pyroptosis in A549 and H460 cells is of a particular interest, as both cell lines are LKB-null and LKB-null lung cancer is generally unresponsive to immunotherapy [30]. Pyroptosis is accompanied with the release of various damage-associated molecular patterns (DAMPs) and cytokines (e.g., IL1β), which can recruit immune cells and perpetuate the inflammatory cascade in the tissues [31, 32]. A recent study showed that initiating pyroptosis in tumor cells led to robust infiltration of immune cells and cytotoxic lymphocyte-mediated tumor cell killing [33]. Another study indicated that nanoparticle-delivered active GSDMD sensitized mammary tumors to anti-PD1 therapy and pyroptosis of less than 15% of tumor cells was sufficient to clear entire tumor grafts by anti-PD1 therapy [34]. Hence, we speculate that pyroptosis caused by HOXC8 knockdown in LKB-null lung cancer cells could simultaneously kill cancer cells and convert non-immunogenic tumor to immunogenic one. In future study, we will investigate the effect of HOXC8 siRNAs on antitumor immunity in LKB-null lung cancer models including patient-derived organoids and immunocompetent mice. Especially, we will investigate the potential of using lipid-conjugated or nanoparticle-delivered HOXC8 siRNA to boost the efficacy of immune checkpoint blockers such as anti-PD1 and anti-PD-L1 in LKB-null lung cancer models.

## Supplementary information

Supplementary Materials

Uncropped gels

## Data Availability

All data generated or analyzed during this study are included in this published article [and its supplementary information].
