# Supplementary material for: HOXC8 impacts lung tumorigenesis by preventing pyroptotic cell death through the suppression of caspase-1 expression
Source: Cell Death Dis. 2025 Jul 23;16(1):552. doi: 10.1038/s41419-025-07867-8 (PMC12287344; doi:10.1038/s41419-025-07867-8)
Supplement: Supplementary file 1 — Supplementary Materials [file 41419_2025_7867_MOESM1_ESM.pdf]

Supplemental Figure S1

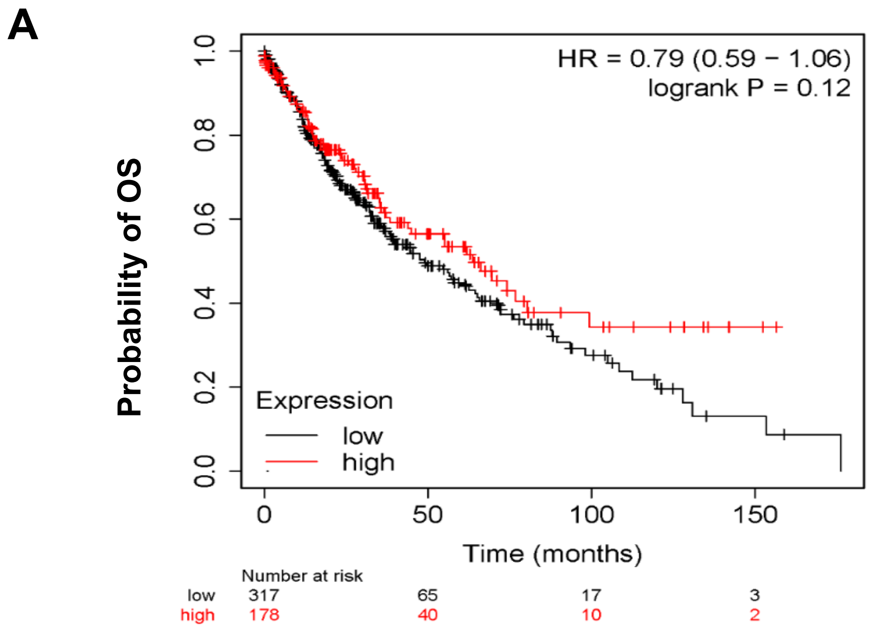

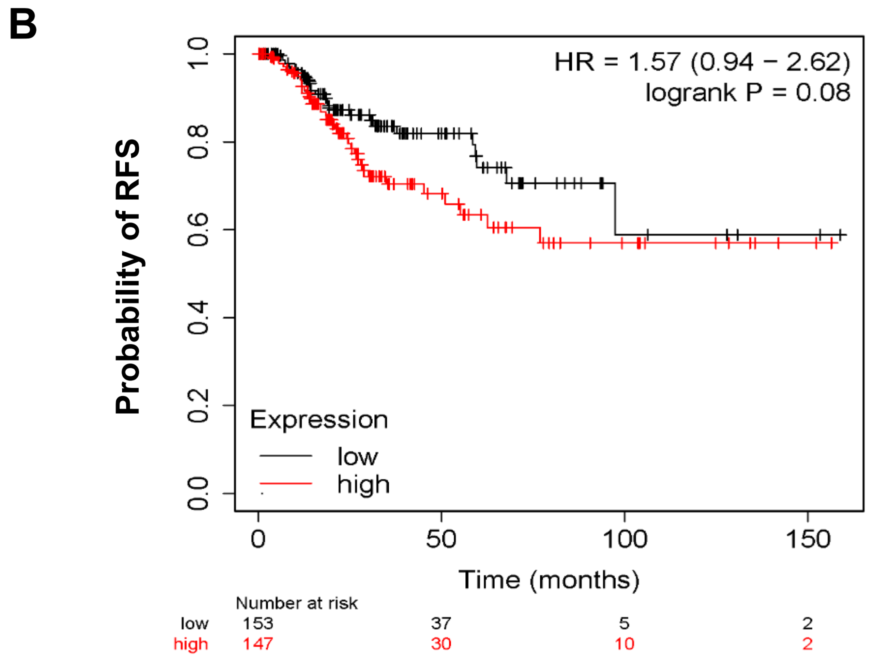

Figure S1. Correlation between HOXC8 expression and survival of LUSC patients. ***A.*** Kaplan-Meier overall survival plots of HOXC8. ***B.*** Kaplan-Meier recurrence-free survival plot of HOXC8.

Supplemental Figure S2

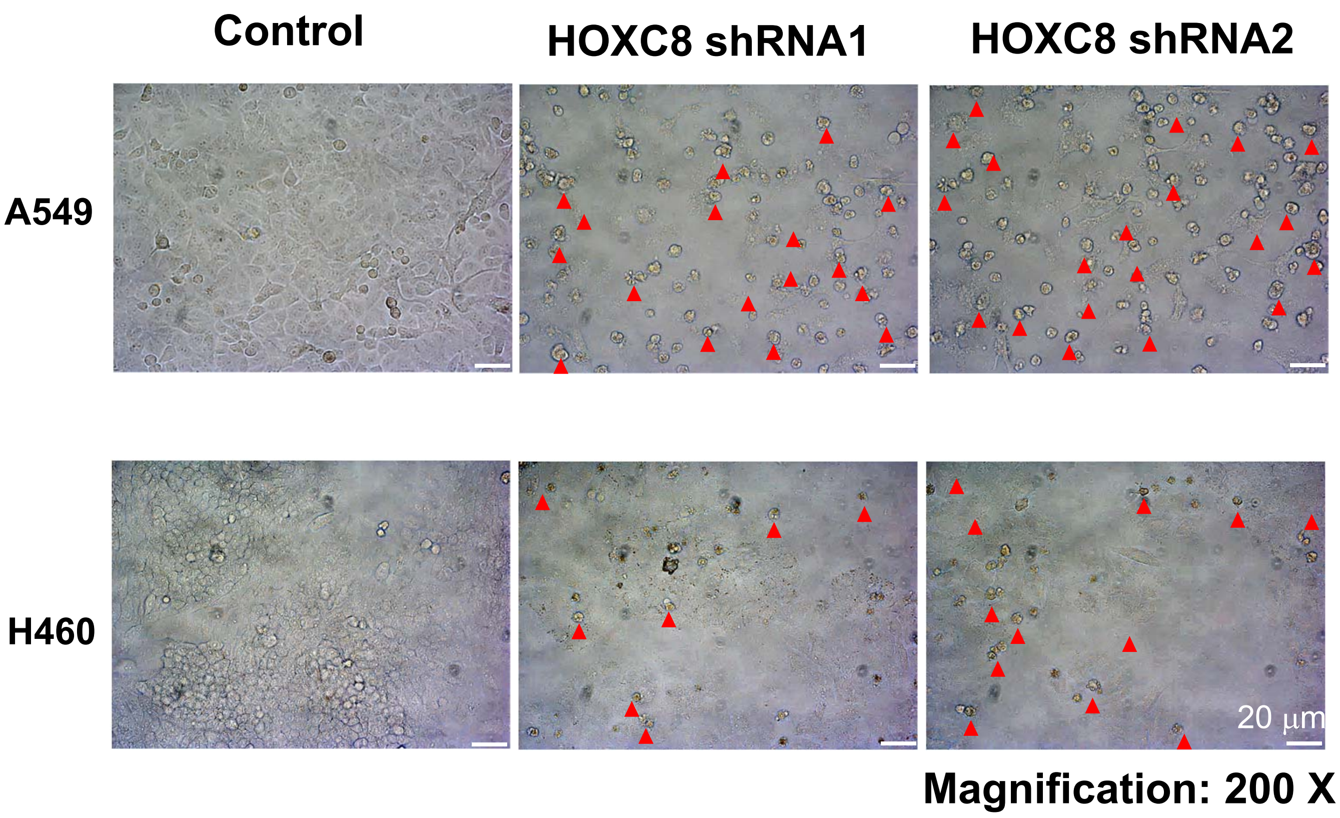

Figure S2. HOXC8 knockdown leads to dramatic cell death. A549 and H460 cells were infected with lentiviral vector containing Scrambled sequence (Control) or HOXC8 shRNA for 4 days followed by visualization under a phase-contrast light microscope. Arrow heads indicate dead cells. Scale bar= 20 μm.

Supplemental Figure S3

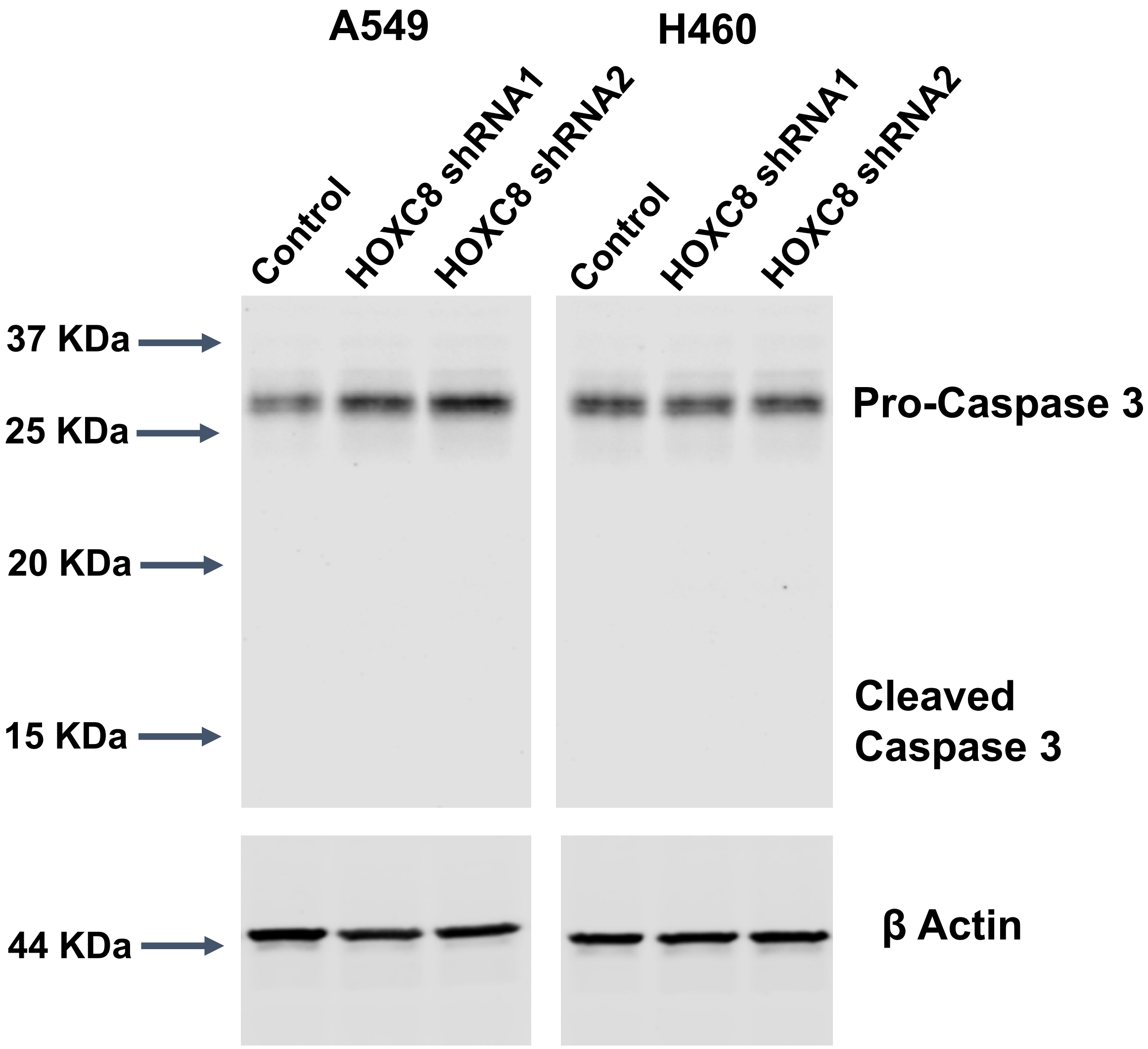

Figure S3. HOXC8 knockdown does not kill cells through apoptosis. A549 and H460 cells were infected with lentiviral vector containing Scrambled sequence (Control) or HOXC8 shRNA for 4 days followed by western blotting to detect caspase-1 and β Actin.

Supplemental Figure S4

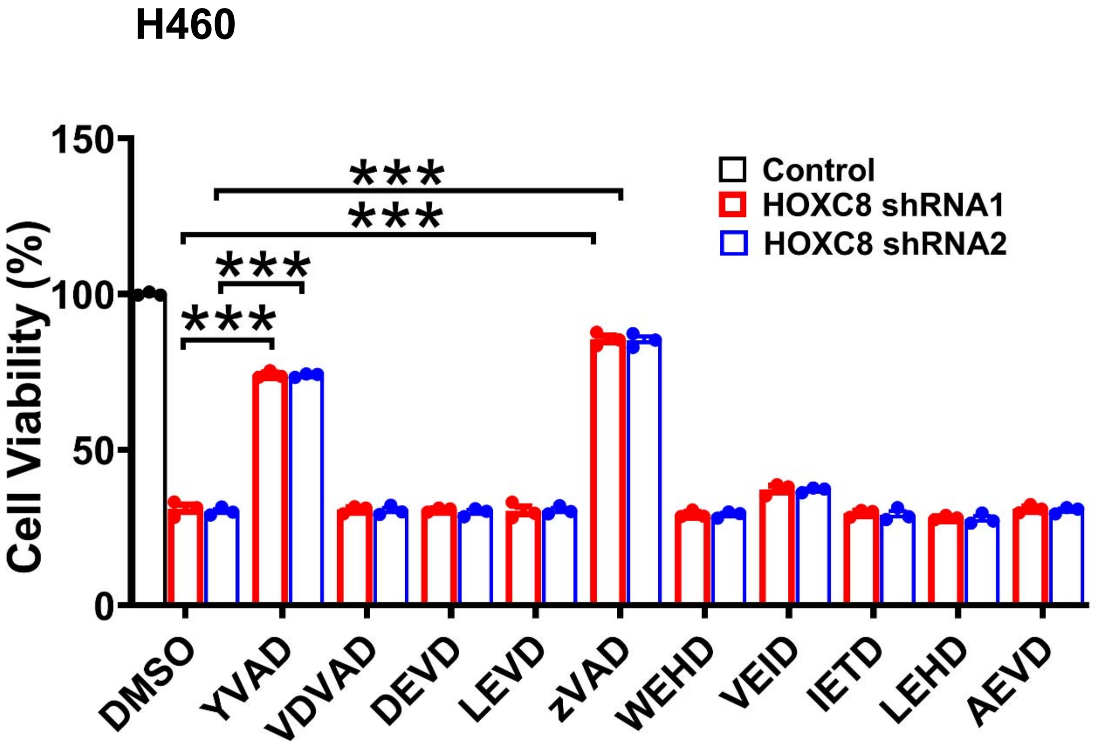

Figure S4. Knockdown of HOXC8 induces caspase-1-mediated cell death in LUAD cells. H460 cells were infected with lentiviral vector containing Scrambled sequence or HOXC8 shRNA for 2 days and then cultured in the presence of vehicle (DMSO) or 10μM inhibitor of each individual caspase for 2 days followed by an MTT assay to analyze cell viability. Data are means ± SEM. ***, $P < 0.001$.

Supplemental Figure S5

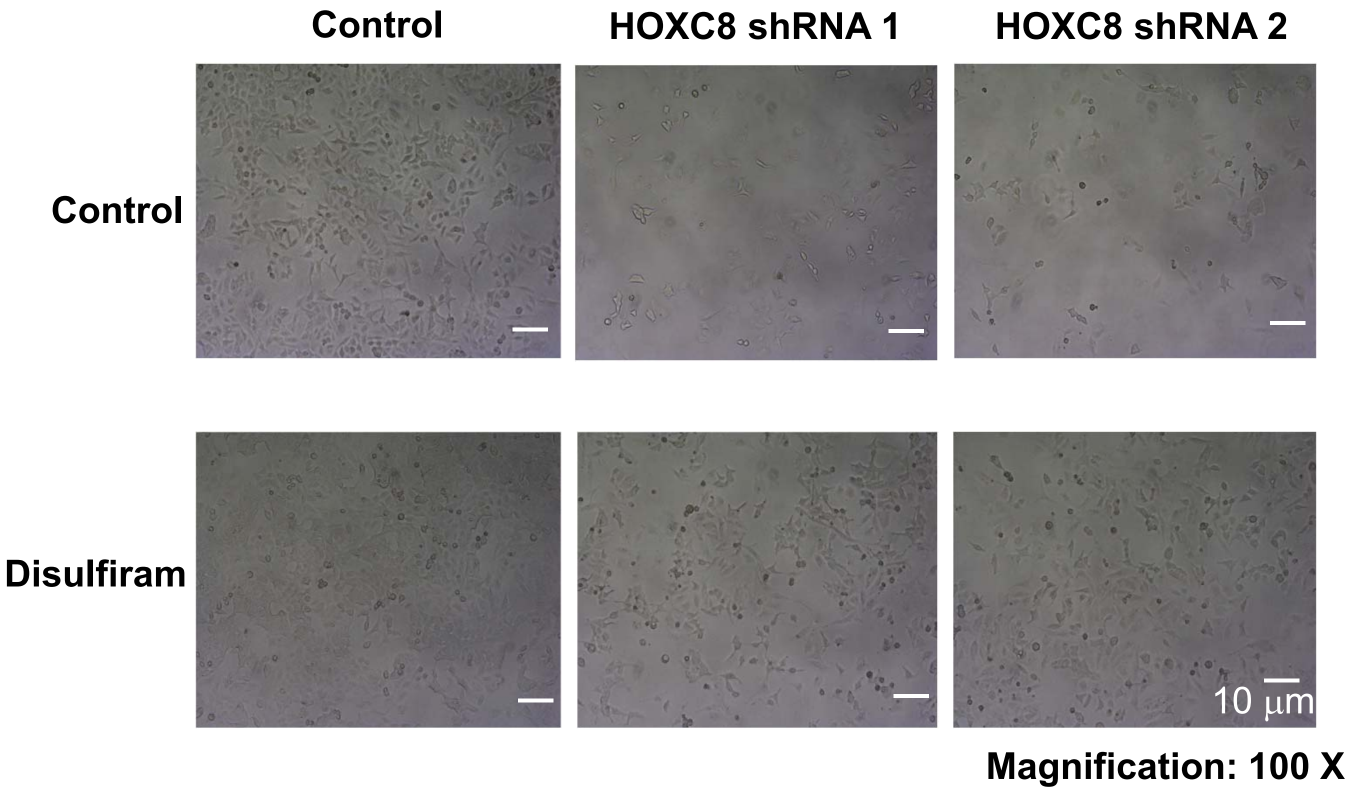

Figure S5. Disulfiram prevent cell death led by HOXC8 knockdown. A549 cells were infected with lentiviral vector containing Scrambled sequence (SC) or HOXC8 shRNA for 2 days followed by treatment with/without Disulfiram. Cells were visualized under a phase-contrast light microscope. Scale bar = 10 mm.

Supplemental Figure S6

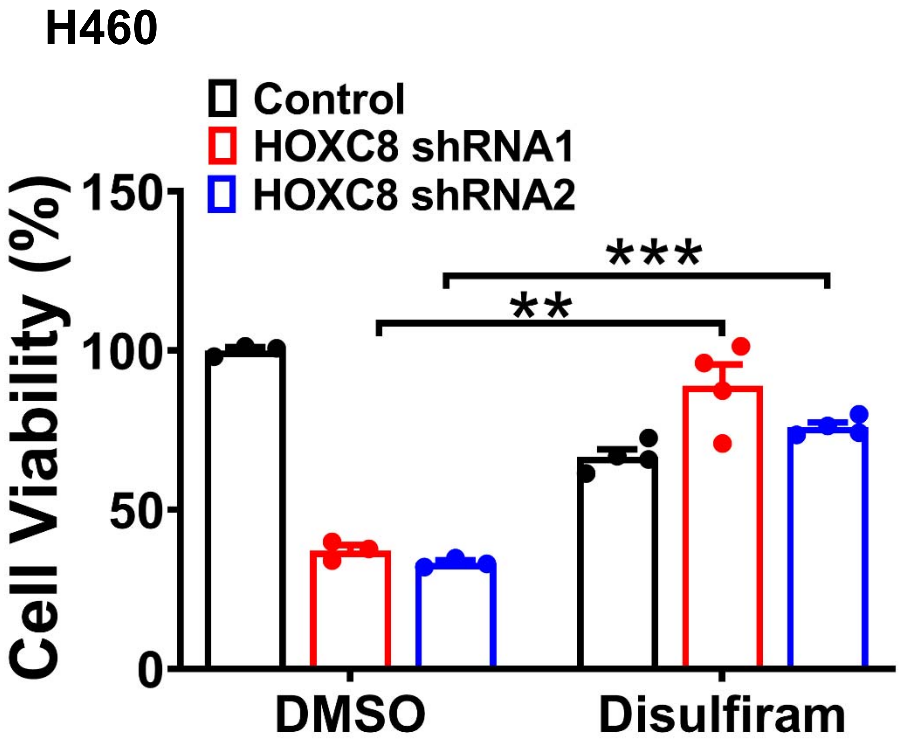

Figure S6. Effect of Disulfiram on cells death induced by HOXC8 knockdown. H460 cells were lentivirally transduced with either Scrambled sequence (control) or HOXC8 shRNA for 2 days and then culture in the presence of vehicle (DMSO) or 10μM disulfiram for another 2 days, followed by an MTT assay to analyze cell viability. Data are means ± SEM. **, $P < 0.01$; ***, $P < 0.001$.

Supplemental Figure S7

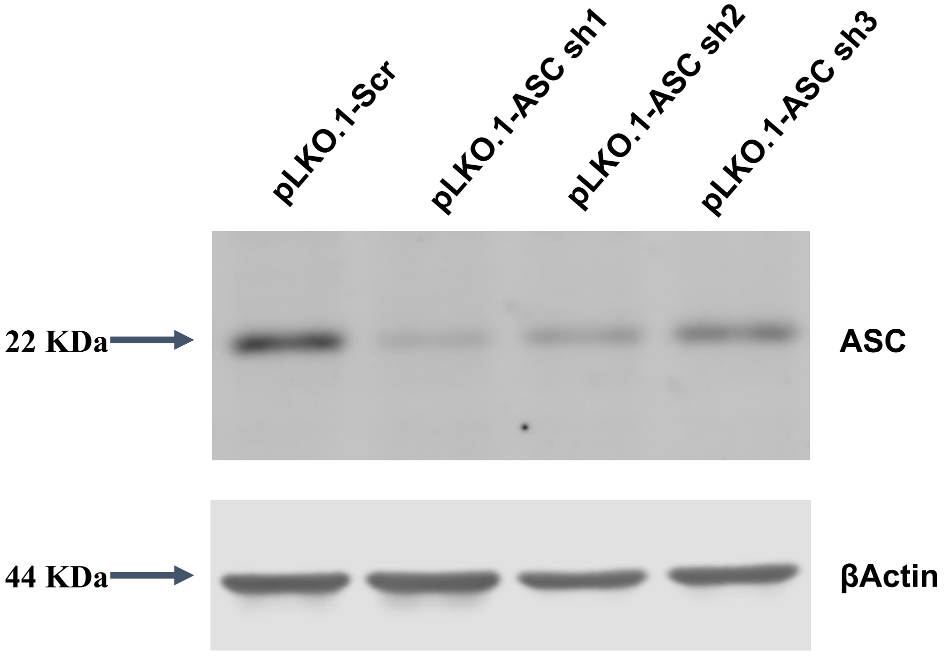

Figure S7. Knockdown of ASC in A549 cells. A549 cells were lentivirally transduced with ASC shRNA for 3 days followed by western blotting to detect ASC and βActin with the respective antibodies.

Supplemental Figure S8

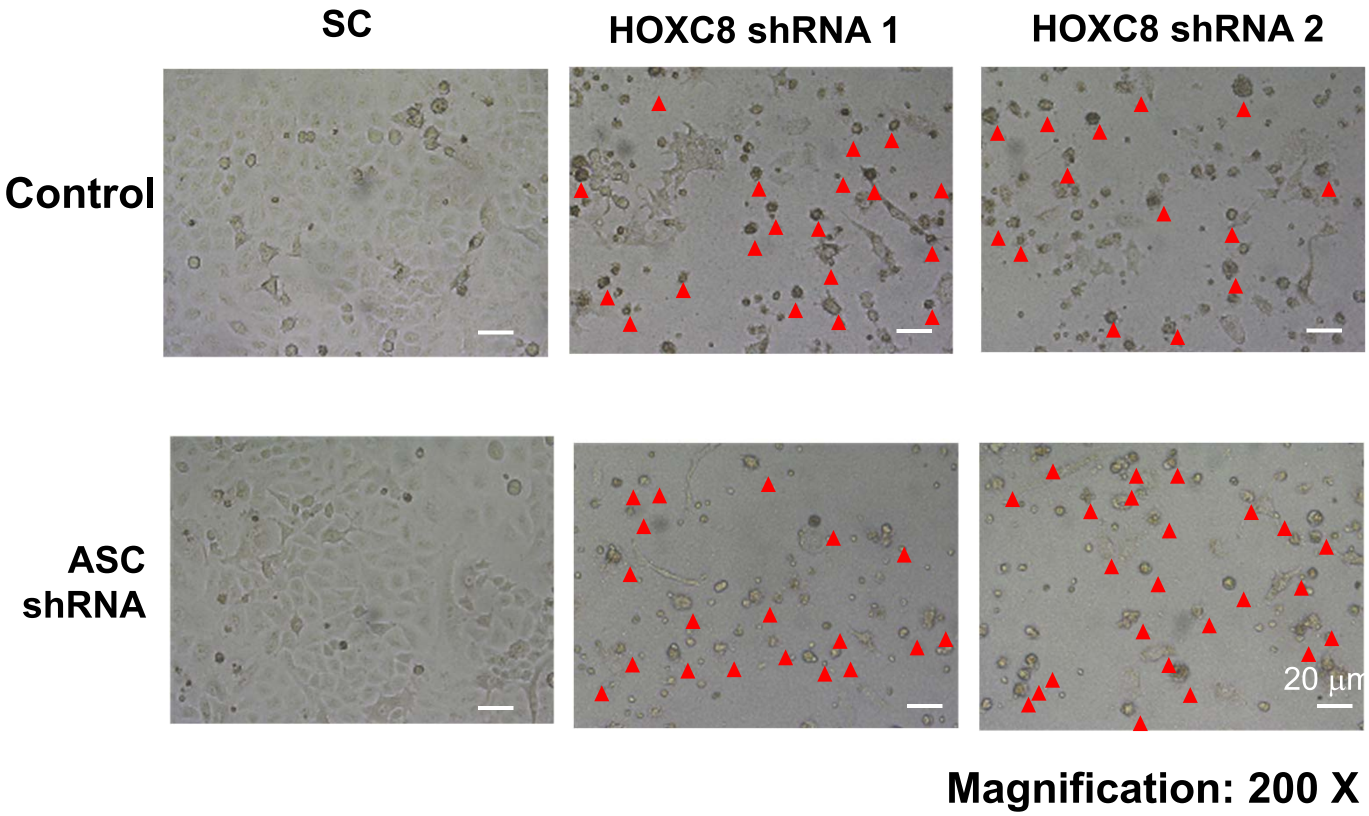

Figure S8. ASC depletion does not prevent cell death led by HOXC8 knockdown. A549 cells were infected with lentiviral vector containing Scrambled sequence (SC) or HOXC8 shRNA for 2 days followed by infection with Scrambled sequence or ASC shRNA for 2 days. Cell morphology were visualized under a phase-contrast light microscope. Arrowheads indicate dead cells. Scale bar= 20 mm.

Supplemental Figure S9

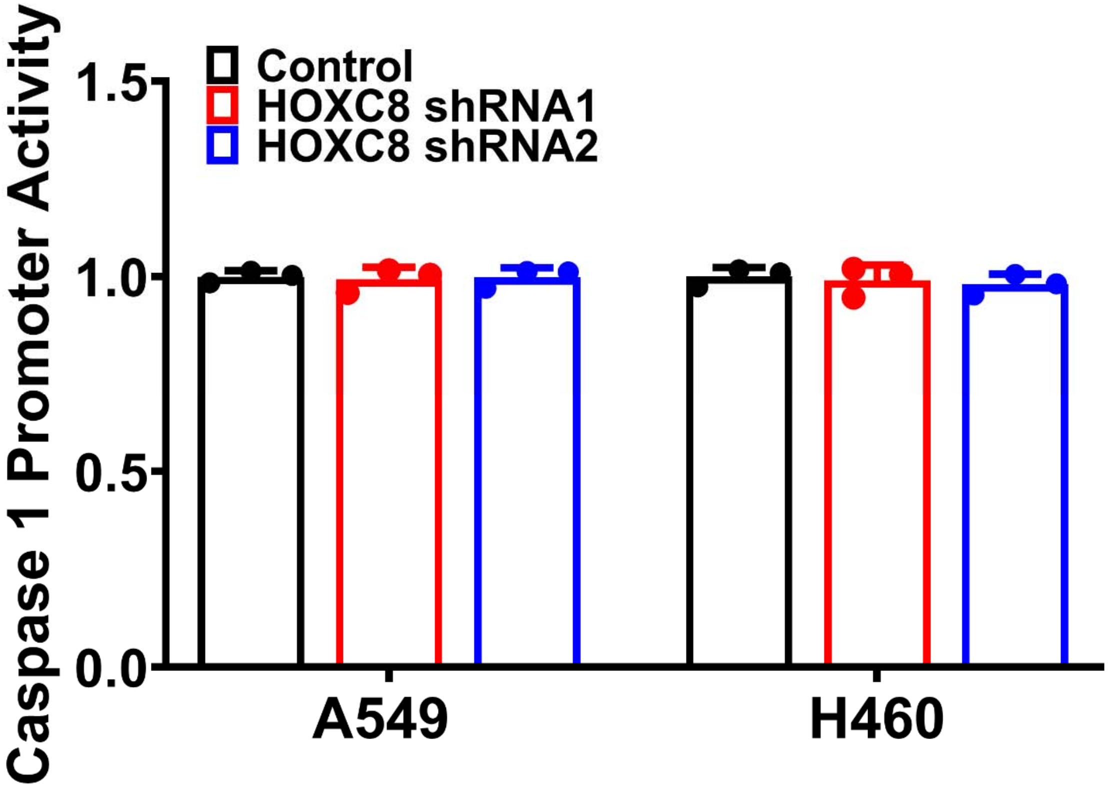

Figure S9. Effect of HOXC8 knockdown on CASP1 promoter activity. A549 and H460 cells were first transduced with HOXC8 shRNA for 3 days then transfected with firefly luciferase reporter plasmid containing caspase-1 promoter for another day. PGK-R/Luc plasmid was included during transfection. The CASP1 promoter activity was calculated by dividing the firefly luciferase activity with Renilla luciferase activity. . Data are means ± SEM.

Supplemental Figure S10

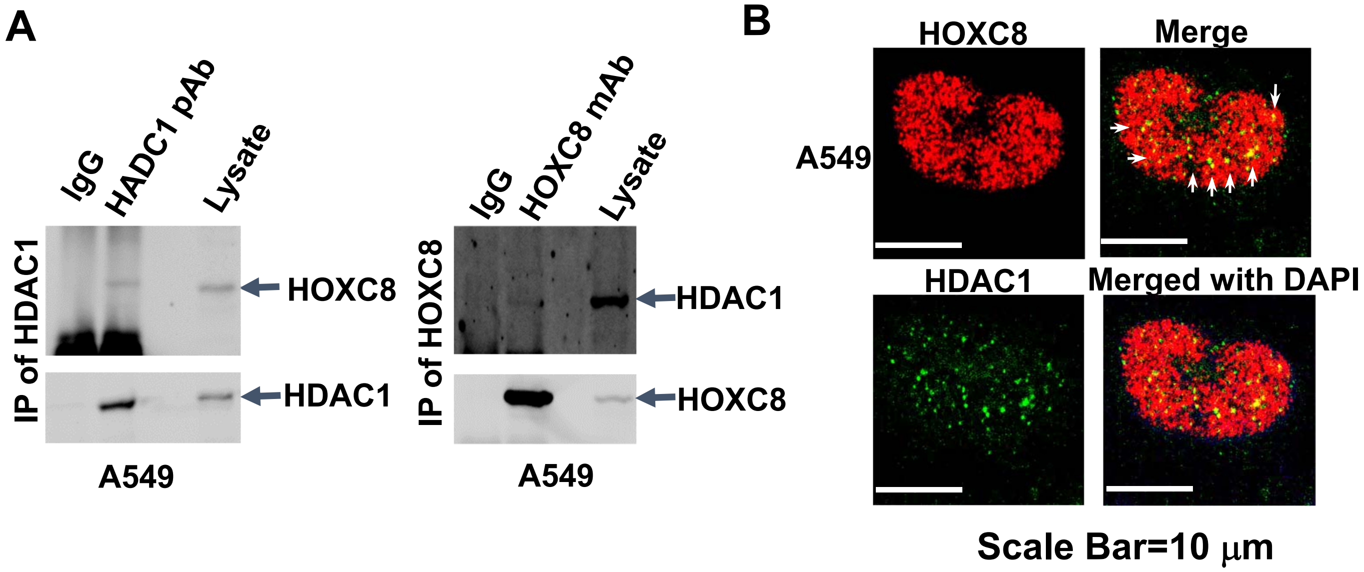

Figure S10. Interaction of HOXC8 and HDAC1 in lung cancer cells. ***A.*** A549 cells were immunoprecipitated with either HDAC1 or HOXC8 antibody and the immunoprecipitates were subjected to western blot to detect HOXC8 or HDAC1 respectively. All blots derived from the same experiment and were processed in parallel. ***B.*** A549 cells were subjected to immunofluorescence staining to detect HOXC8 and HDAC1. Data are the representative of six independent experiments.

Supplemental Figure S11

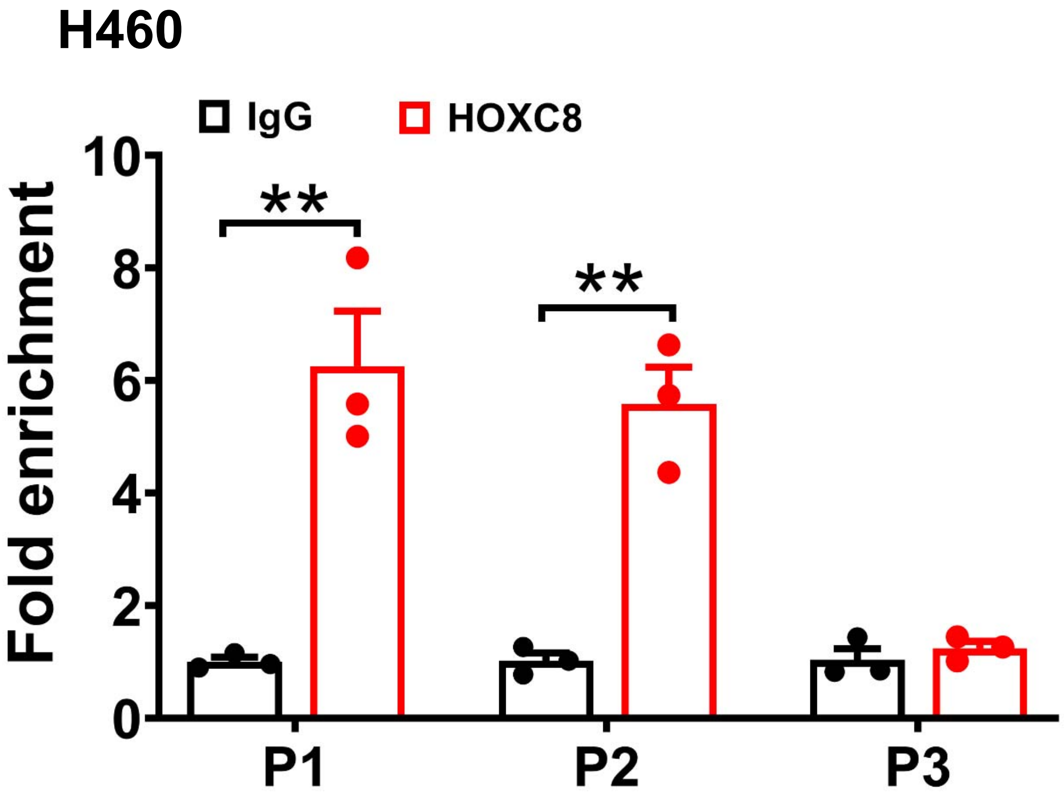

Figure S11. Occupancy of HOXC8 in CASP1 promoter. H460 cells were subjected to ChIP with IgG or HOXC8 mAb followed by qPCR to analyze the occupancy of HOXC8 in P1, P2 and P3 regions of caspase-1 promoter. Data are means ± SEM. **, $P < 0.01$.

Supplemental Figure S12

AGAGACAGACCATATCACCAGCCTTCATGTTTGTCTTTCCCTCTTGAATGTGAGACCAAAGGGGAAAGGA
CCATGATATGTTTTGACTGAACCTCTGGCACCTAGAGCAATGTCGTCCGTATATGTTTGCTAAATGAATG
ACTAGATGCACTGCTTGAGGATTCTAACAAAAGATAAACTTAGCTAAATAATCTGCAAATAATCATCCTT
GATTATTTTTCTAACCTTAAACATTTGTATTAATTATAATAATATCACTCATTATTTGCTTACCATTTGC
CAGGCTATGTTCCATGCATTTTCCACATTTTCTCTCACTTAATGCTCAAAACTGTGTAGGTACATACTAT
TAATAAAATTATTTCACATACAGAGGAACCAATGCATAGGGAAGTTCATTATCTTGCCAAGGAAGGGAAG
TTTAAAATGCTAAGTTCGACATTCTCATTCCAGAGCCTATGCTCTGGGCCTCTACAATGCAATTTCCCTC
AAAAATAAACCCTCCAGGATGGGTGGGTGGCTCATGCCTGTAATCCTAGCAATTTGGGAGACCAAGGTGG
GCACATTGCTTGAGCCTGGGAGCTGAAGACAAGCCTAGGAAACACAAGGAGACCCTGTCTTTACAAGAGA
TACAAATATTAGCTGGCCATGGTGGCATGTGCCAGTAATTGCAGCTACTCGGGAGGCTGAGTTGGAAGAA
TCATCTGAGCCTGGGAAGTGGAGGCTGCAGTGAGCCAAGGTCAAATAACTGCATTCCGGCCTGCACGACA
GAATGATACCCTGTATCAGAAAAAAAAAAAAACAGAAAGAAAAAAGCAATGTGAAAAGAAGGACATTAAA
TAAGAAAAGCTTTAATTCTGGTGAAAAATGTTTCTATTTCTTAAAGCAAAAATGTTTCACAGGGACATAC
ATTACCCACAACTTTCCCAAGAGCTATGAGGTGGGGTCAGGGGAACAGTGGTTCACATACTCCAAAGGTT
GTGTTACTTTTCTCCGTCAAGCTTTCTAATGATTGAGAAACTCTTCACTGTGTGAATTTAATTGTACACA
TACTTTTTCAATCCTGTCTTACTCTCCAAGCCCCATTCCCCTCCTACCCTGATCTATCCAAGGGCTGGTG
AAGAAAATTTCCCATGGATACACTACCTGATGCAGGCTACAGTTCTGATTCTTTAATGGGAAGAAAAATA
AAGACATGCATATGCATGCACAGTGAGTATTTCCCAATACATGTACAGGCCCTGCCAAAAAGGAAGGCGA
AGCATACTTTCAGTTTCAGTCACACAAGAAGGGAGGAGAGAAAAGCCATGGCCGGTGAGTCTTTTACTGT

+1 +32

P3:-1170- 996 (175 bp)

P2:-955-786 (170bp)

P1:-421-272 (150bp)

Figure S12. Sequence of CASP1 promoter. Highlighted areas are P1, P2 and P3 regions.

Supplemental Figure S13

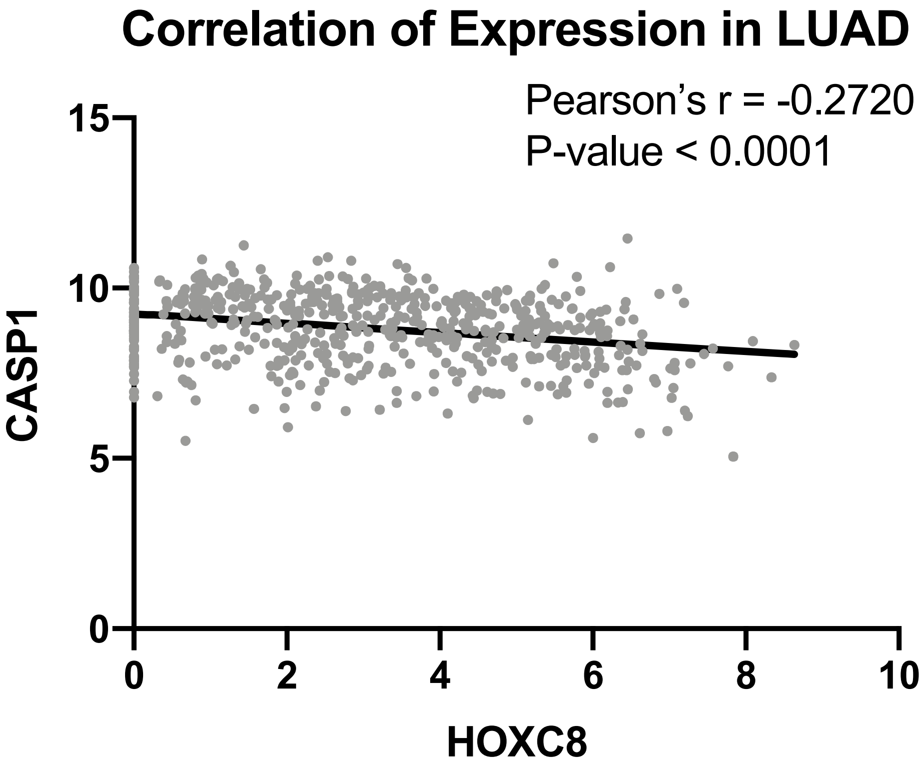

Figure S13. Correlation between CASP1 and HOXC8 expression in LUAD. TCGA dataset was analyzed for Pearson's r value.

Supplemental Figure S14

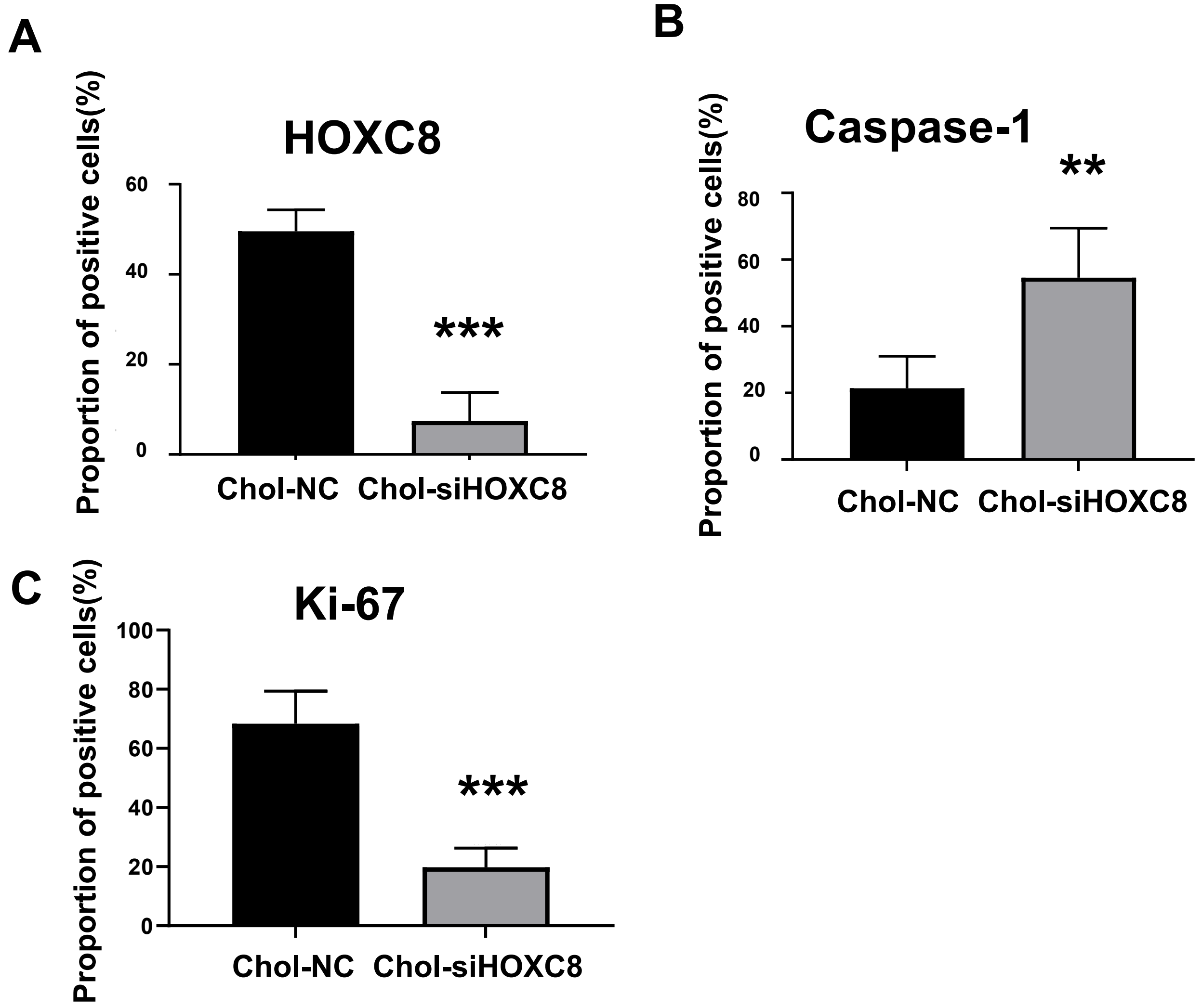

Figure S14. Quantitative Analysis of HOXC8, Ki67, and Caspase-1 staining in tumor tissues excised from Chol-NC and Chol-siHOXC8-treated mice. Tumor sections were subjected to IHC and cells with positive staining were counted under microscope. At least 100 cells were counted for each section. Statistical analysis was performed using Student's t-test and data are presented as mean ± SEM (**, $P < 0.01$, ***, $P < 0.001$).

Supplemental Figure S15

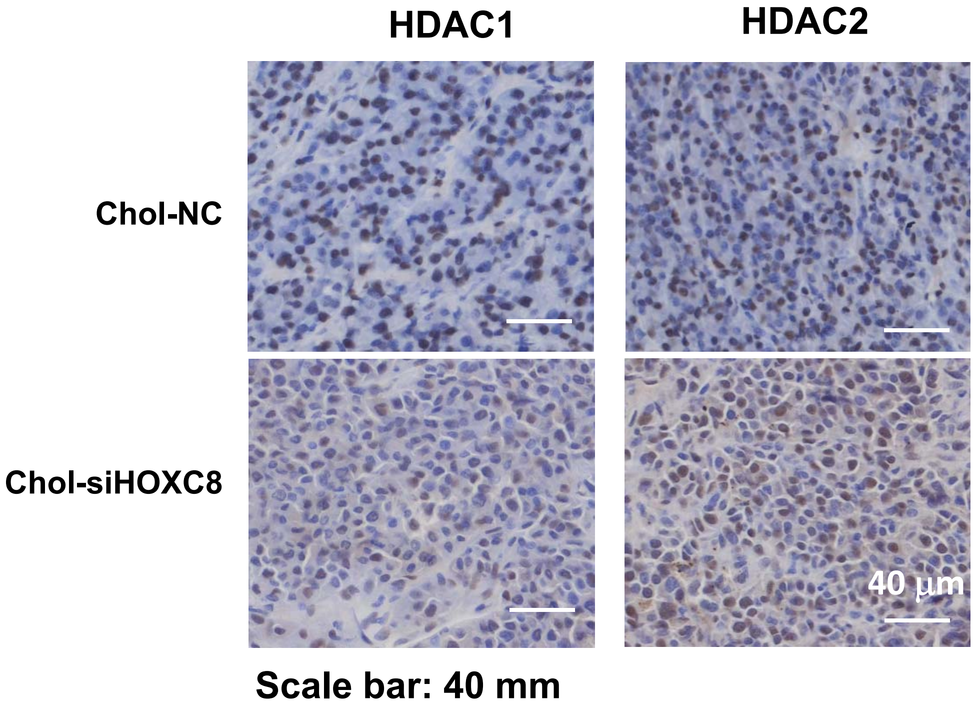

Figure S15. HDAC1 and HDAC2 staining in tumor tissues excised from Chol-NC and Chol-siHOXC8-treated mice. Tumor sections were subjected to IHC and visualized under microscope. Representative pictures of IHC staining on HOXC8, caspase-1, Ki67 and cleaved caspase-3 in tumor tissues.

Supplemental Figure S16

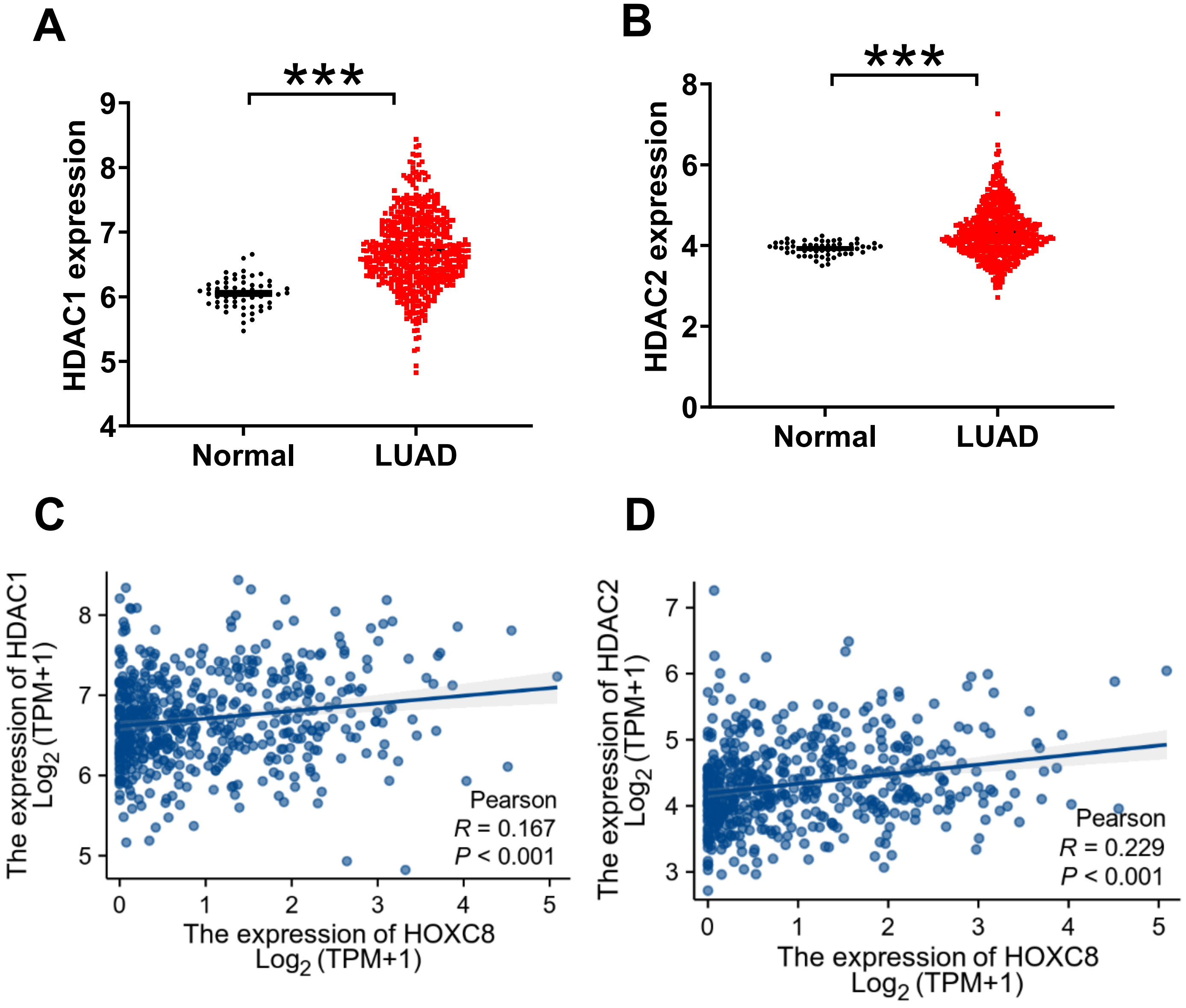

Figure S16. HDAC1 and HDAC2 expression in LUAD. ***A***. Level of HDAC1 mRNA in normal lung and LUAD tissue. ***, p < 0.001. ***B.*** Level of HDAC2 mRNA in normal lung and LUAD tissue. ***, p < 0.001. ***C***. Correlation between HDAC1 and HOXC8 mRNA in LUAD. ***D***. Correlation between HDAC2 and HOXC8 mRNA in LUAD.

Table S1. Primer Sequences

Primer	Sequence 5′ to 3′	Description
Casp-1	Fwd: GCTTTCTGCTCTTCCACACCA<br>Rev: AAATGAAAATCGAACCTTGCGGA	qRT-PCR
β-Actin	Fwd: CCTCGCCTTTGCCGATCC<br>Rev: CTCGTCGCCCACATAGGAAT	qRT-PCR
P1	Fwd: TTCTGGTGAAAAATGTTTCTATT<br>Rev: AAAGCTTGACGGAGAAAAGT	ChIP for HA-HOXC8 occupancy at Casp-1 Promoter
P2	Fwd: ATGCTCAAAACTGTGTAGGTA<br>Rev: TGAGGGAAATTGCATTGTAGA	ChIP for HA-HOXC8 occupancy at Casp-1 Promoter
P3	Fwd: GCAATGTCGTCCGTATATGT<br>Rev: CTGGCAAATGGTAAGCAAAT	ChIP for HA-HOXC8 occupancy at Casp-1 Promoter
	Fwd: ACTTTTCTCCGTCAAGCTTTCT<br>Rev: GCTTCGCCTTCCTTTTTGGC	ChIP for HDAC1 occupancy at Casp-1 Promoter
	Fwd: CTGGTGAAGAAAATTTCCCATGGAT<br>Rev: CCCTTCTTGTGTGACTGAAACTG	ChIP for RP-II occupancy at Casp-1 Promoter
