# Supplementary material for: HOXC8 impacts lung tumorigenesis by preventing pyroptotic cell death through the suppression of caspase-1 expression
Source: Cell Death Dis. 2025 Jul 23;16(1):552. doi: 10.1038/s41419-025-07867-8 (PMC12287344; doi:10.1038/s41419-025-07867-8)

Figure 3E

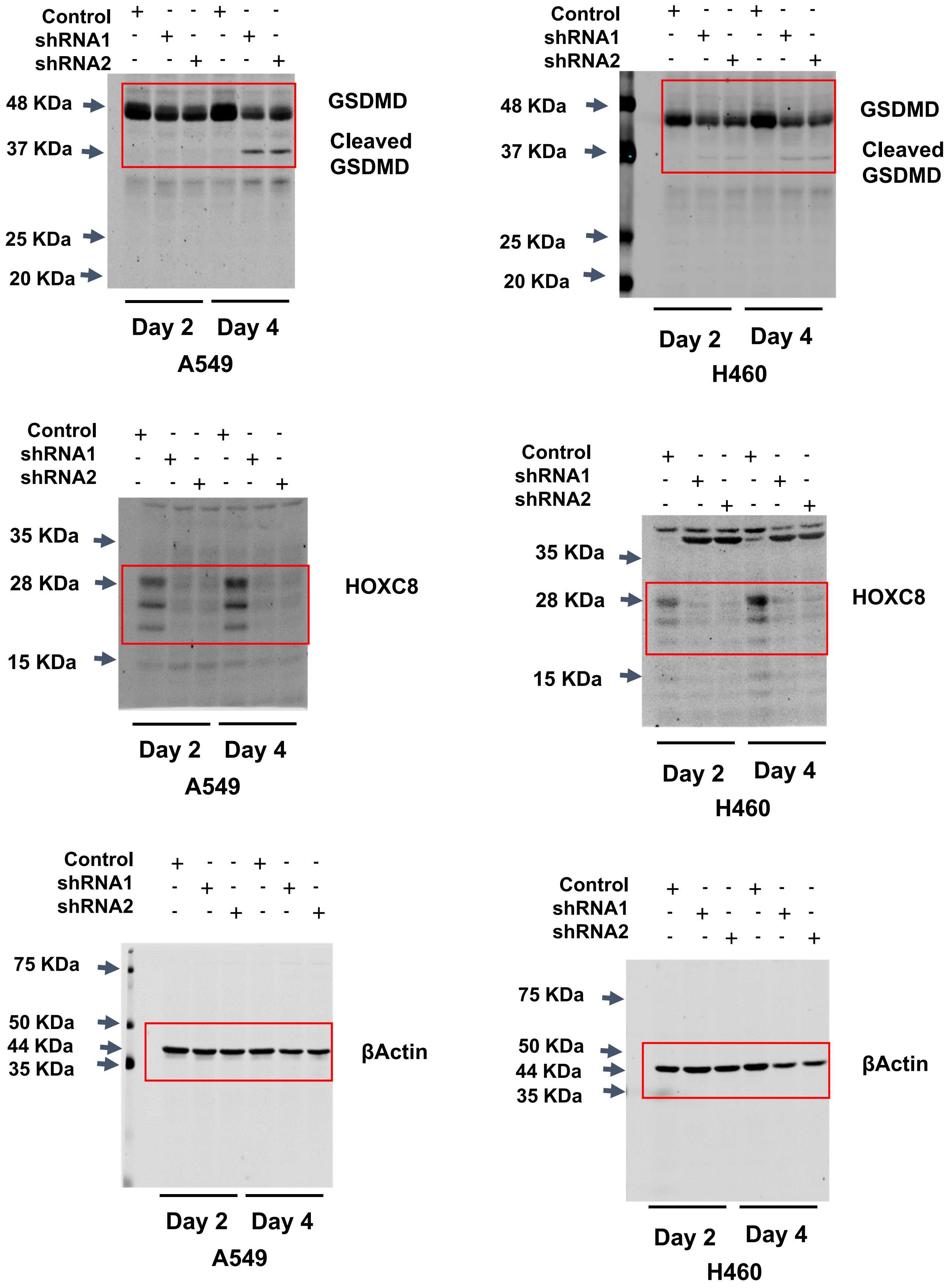
Control + - - + - -
shRNA1 - + - - + -
shRNA2 - - + - - +
48 KDa
37 KDa
25 KDa
20 KDa
GSDMD
Cleaved GSDMD
Day 2
Day 4
A549
Control + - - + - -
shRNA1 - + - - + -
shRNA2 - - + - - +
48 KDa
37 KDa
25 KDa
20 KDa
GSDMD
Cleaved GSDMD
Day 2
Day 4
H460
Control + - - + - -
shRNA1 - + - - + -
shRNA2 - - + - - +
35 KDa
28 KDa
15 KDa
HOXC8
Day 2
Day 4
A549
Control + - - + - -
shRNA1 - + - - + -
shRNA2 - - + - - +
35 KDa
28 KDa
15 KDa
HOXC8
Day 2
Day 4
H460
Control + - - + - -
shRNA1 - + - - + -
shRNA2 - - + - - +
75 KDa
50 KDa
44 KDa
35 KDa
βActin
Day 2
Day 4
A549
Control + - - + - -
shRNA1 - + - - + -
shRNA2 - - + - - +
75 KDa
50 KDa
44 KDa
35 KDa
βActin
Day 2
Day 4
H460

Figure 4 D

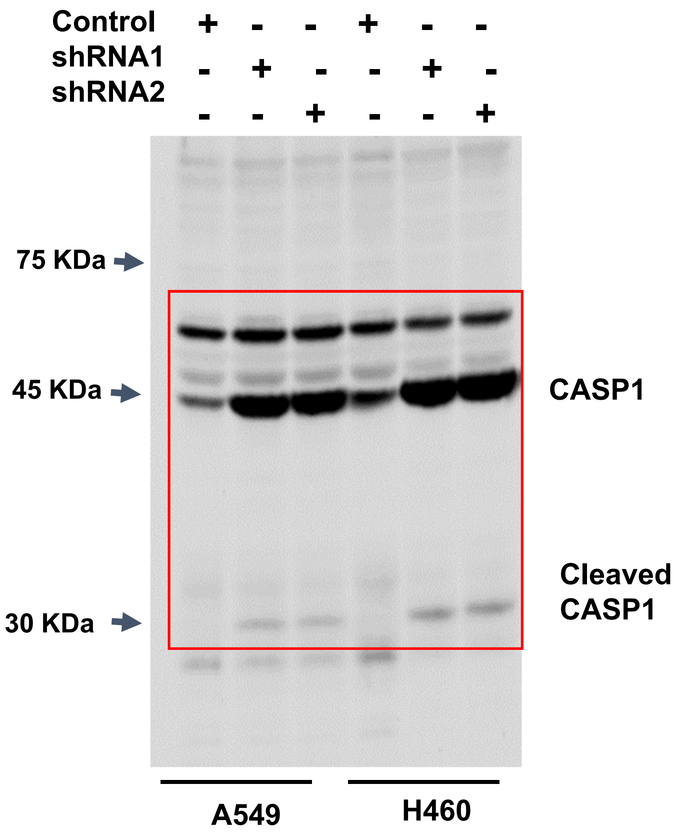
Control + - - + - -
shRNA1 - + - - + -
shRNA2 - - + - - +
75 KDa
45 KDa
30 KDa
CASP1
Cleaved CASP1
A549
H460

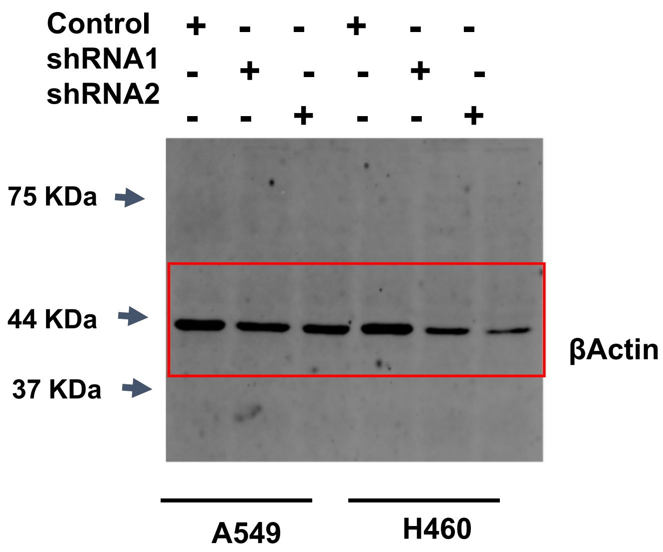
Control + - - + - -
shRNA1 - + - - + -
shRNA2 - - + - - +
75 KDa
44 KDa
37 KDa
βActin
A549
H460

Figure 4 D

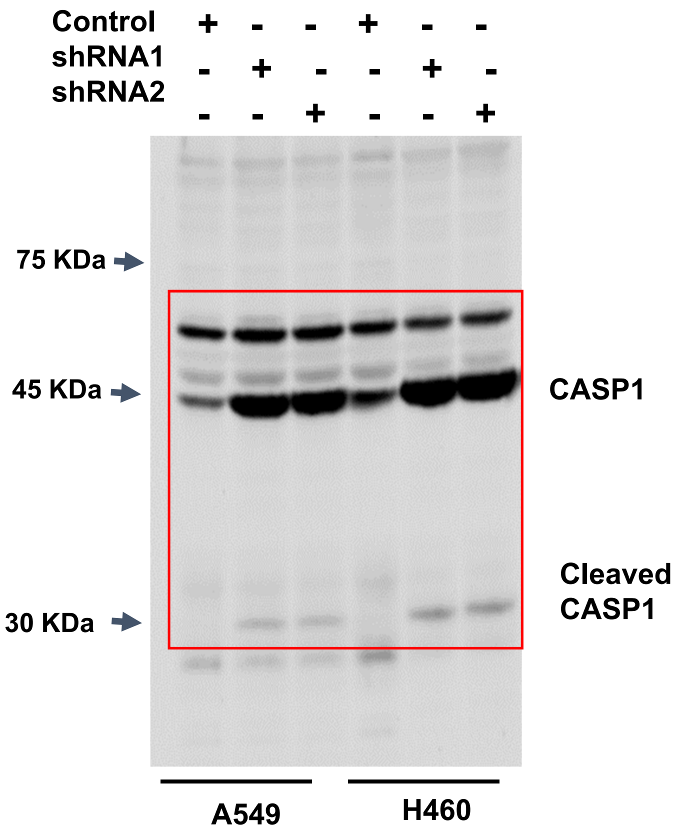

Control + - - + - -
shRNA1 - + - - + -
shRNA2 - - + - - +
75 KDa
45 KDa
30 KDa
CASP1
Cleaved CASP1
A549
H460

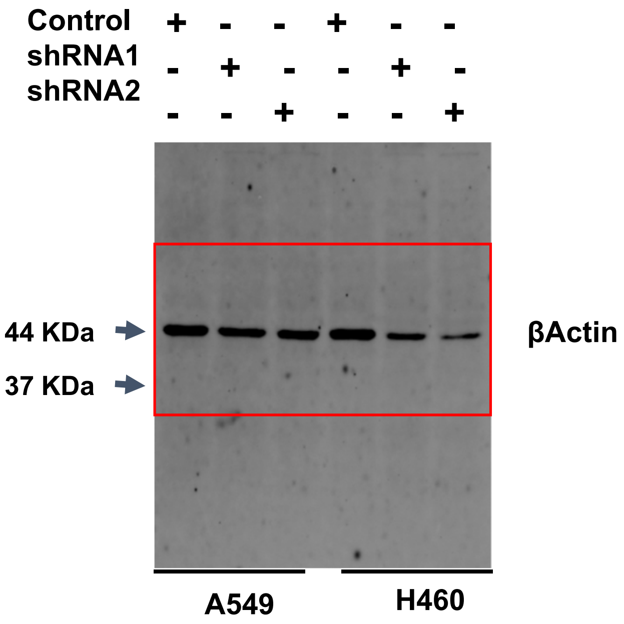

Control + - - + - -
shRNA1 - + - - + -
shRNA2 - - + - - +
44 KDa
37 KDa
βActin
A549
H460

Figure 4E

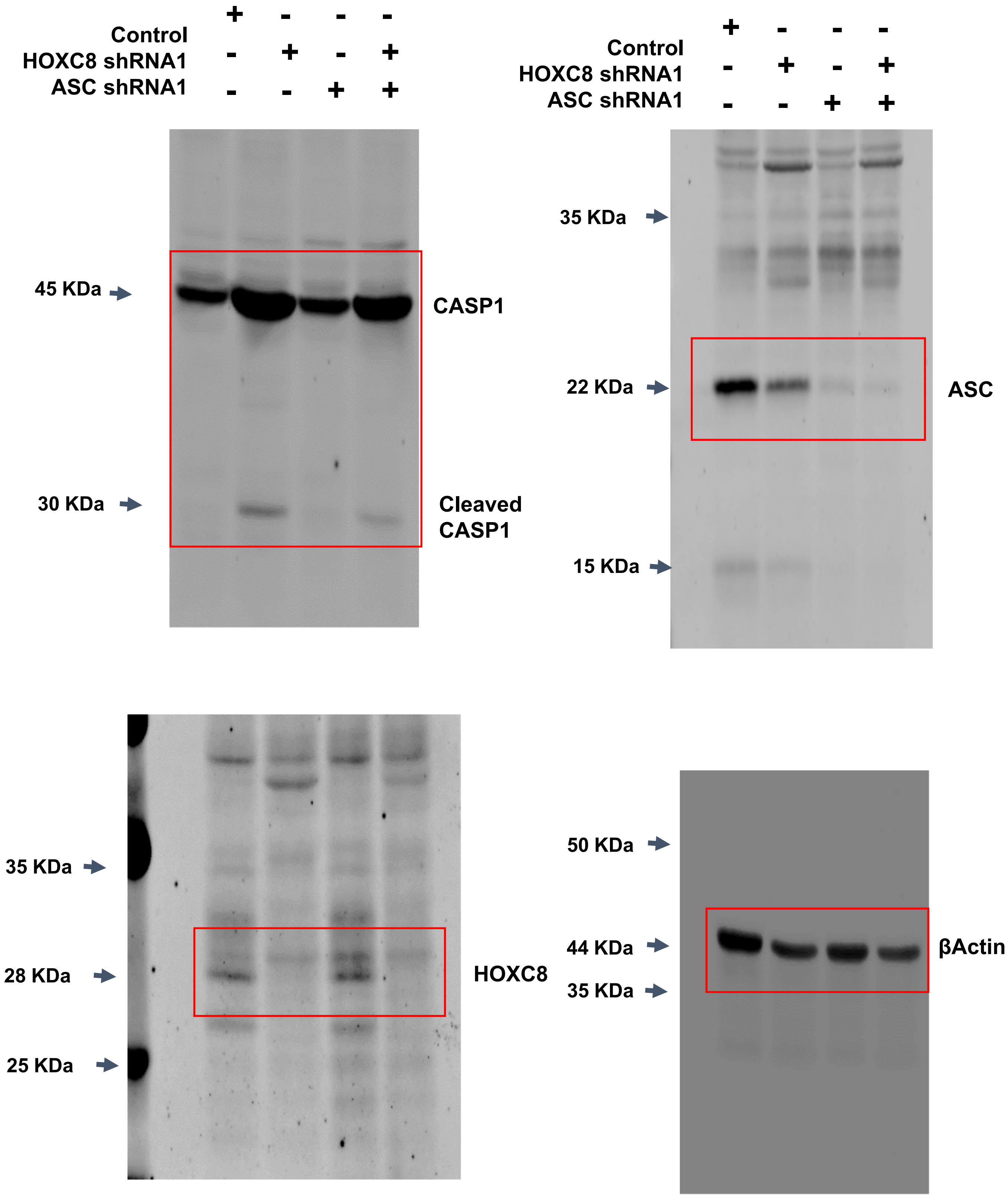

Control
HOXC8 shRNA1
ASC shRNA1
+ - - -
- + - +
- - + +
45 KDa
30 KDa
CASP1
Cleaved CASP1
Control
HOXC8 shRNA1
ASC shRNA1
+ - - -
- + - +
- - + +
35 KDa
22 KDa
15 KDa
ASC
35 KDa
28 KDa
25 KDa
HOXC8
50 KDa
44 KDa
35 KDa
βActin

Figure 4F

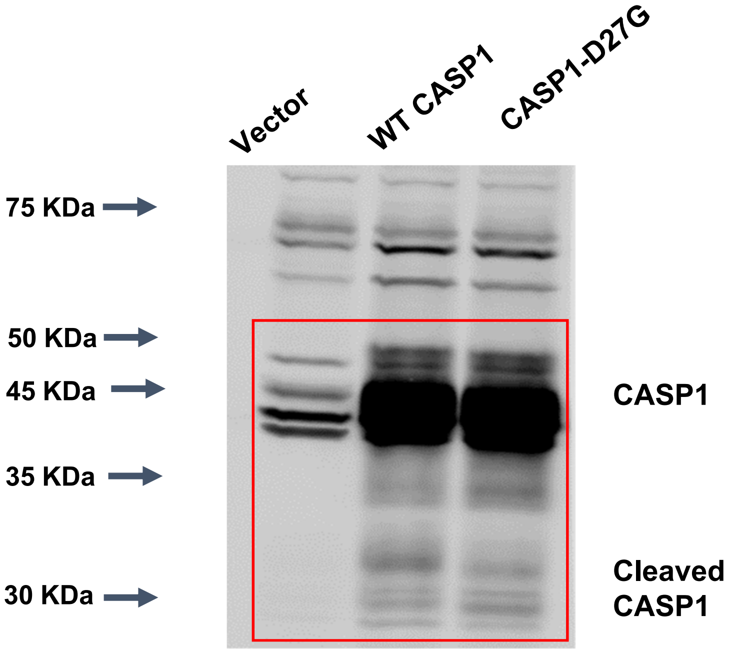

Vector
WT CASP1
CASP1-D27G
75 KDa
50 KDa
45 KDa
35 KDa
30 KDa
CASP1
Cleaved
CASP1

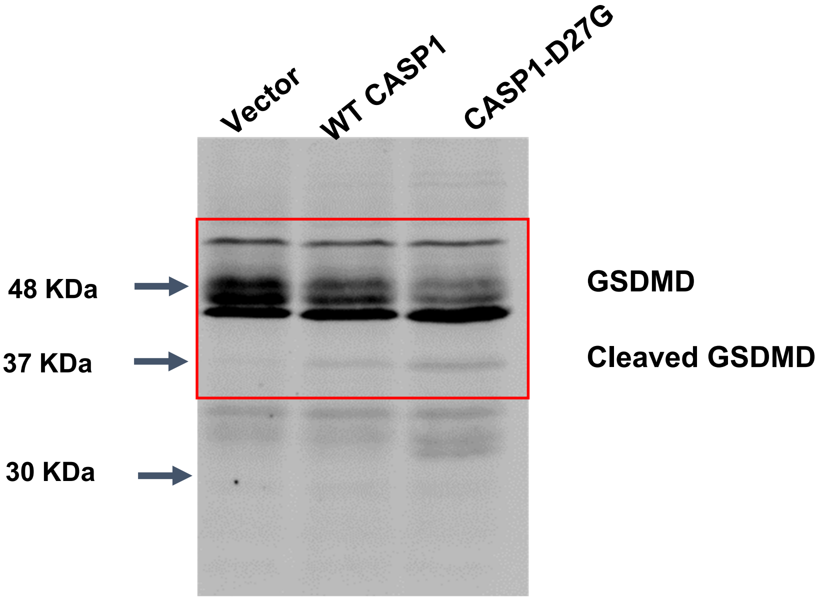

Vector
WT CASP1
CASP1-D27G
48 KDa
37 KDa
30 KDa
GSDMD
Cleaved GSDMD

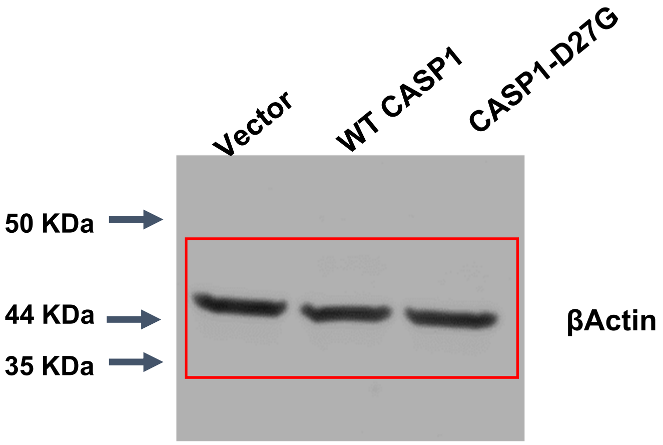

Vector
WT CASP1
CASP1-D27G
50 KDa
44 KDa
35 KDa
βActin

Figure 5C

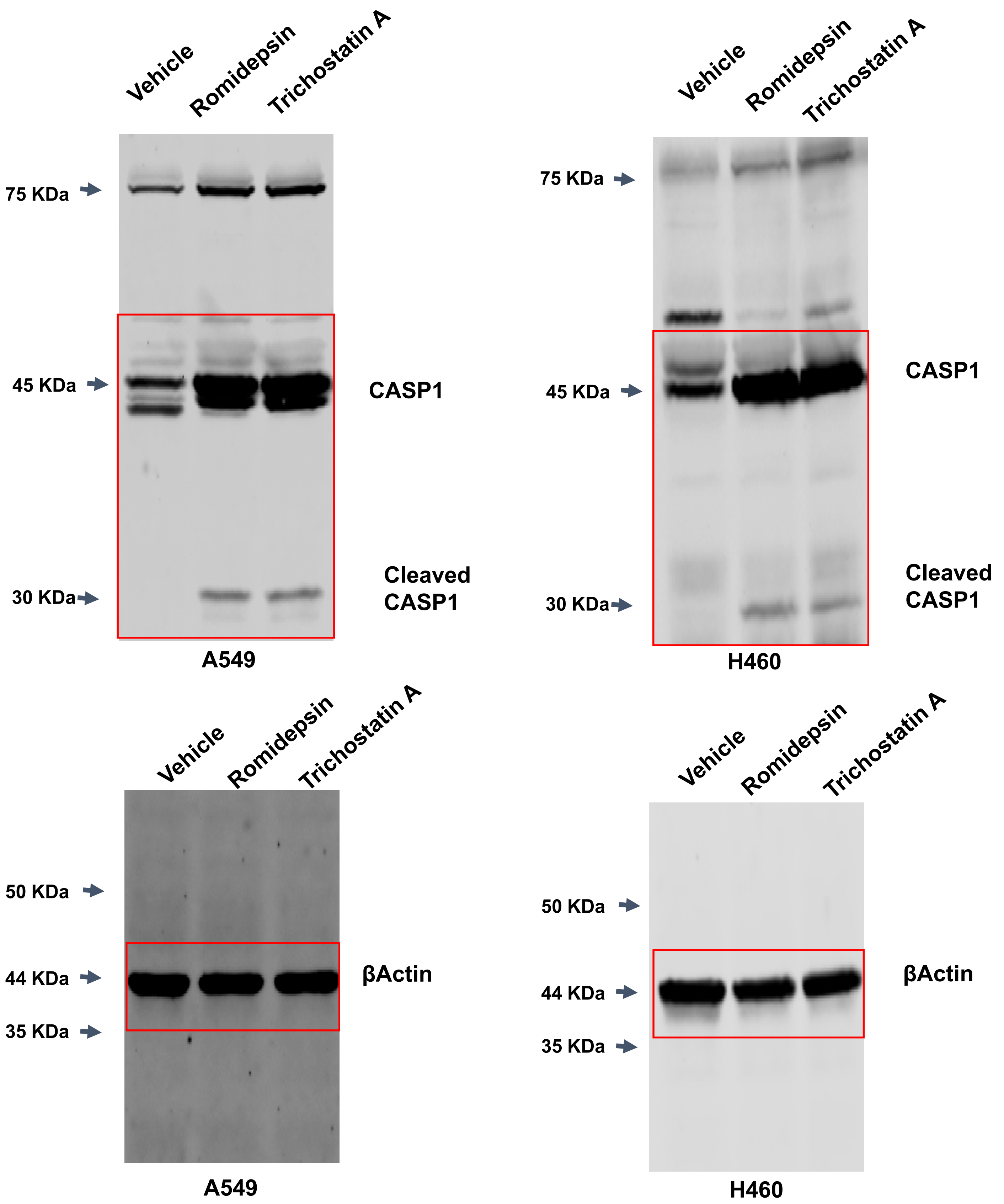

Vehicle
Romidepsin
Trichostatin A
75 KDa
45 KDa
30 KDa
CASP1
Cleaved CASP1
A549
Vehicle
Romidepsin
Trichostatin A
75 KDa
45 KDa
30 KDa
CASP1
Cleaved CASP1
H460
Vehicle
Romidepsin
Trichostatin A
50 KDa
44 KDa
35 KDa
βActin
A549
Vehicle
Romidepsin
Trichostatin A
50 KDa
44 KDa
35 KDa
βActin
H460

Figure 5D

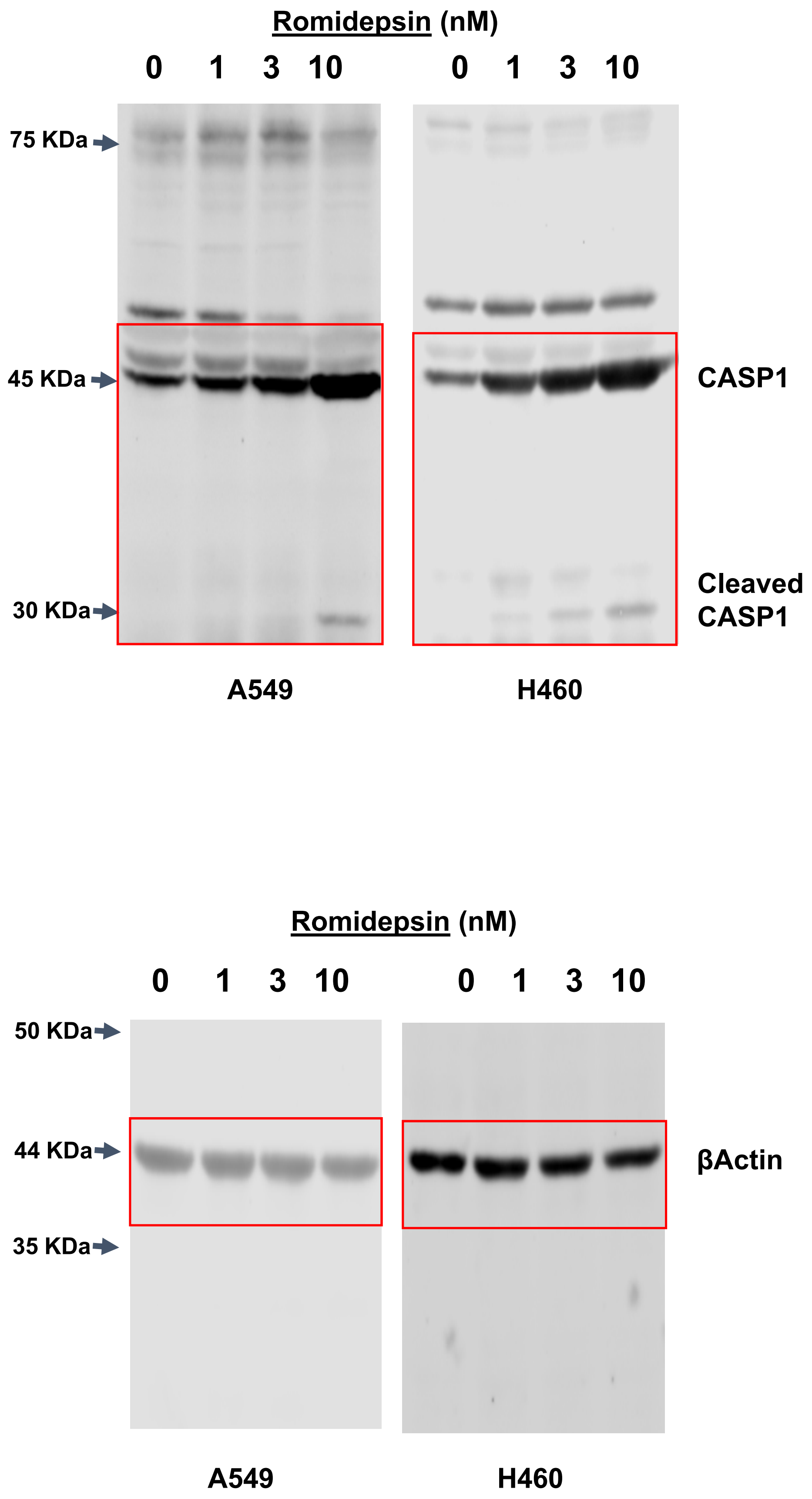
Romidepsin (nM)
0 1 3 10 0 1 3 10
75 KDa
45 KDa
30 KDa
CASP1
Cleaved CASP1
A549
H460
Romidepsin (nM)
0 1 3 10 0 1 3 10
50 KDa
44 KDa
35 KDa
βActin
A549
H460

Figure 6A

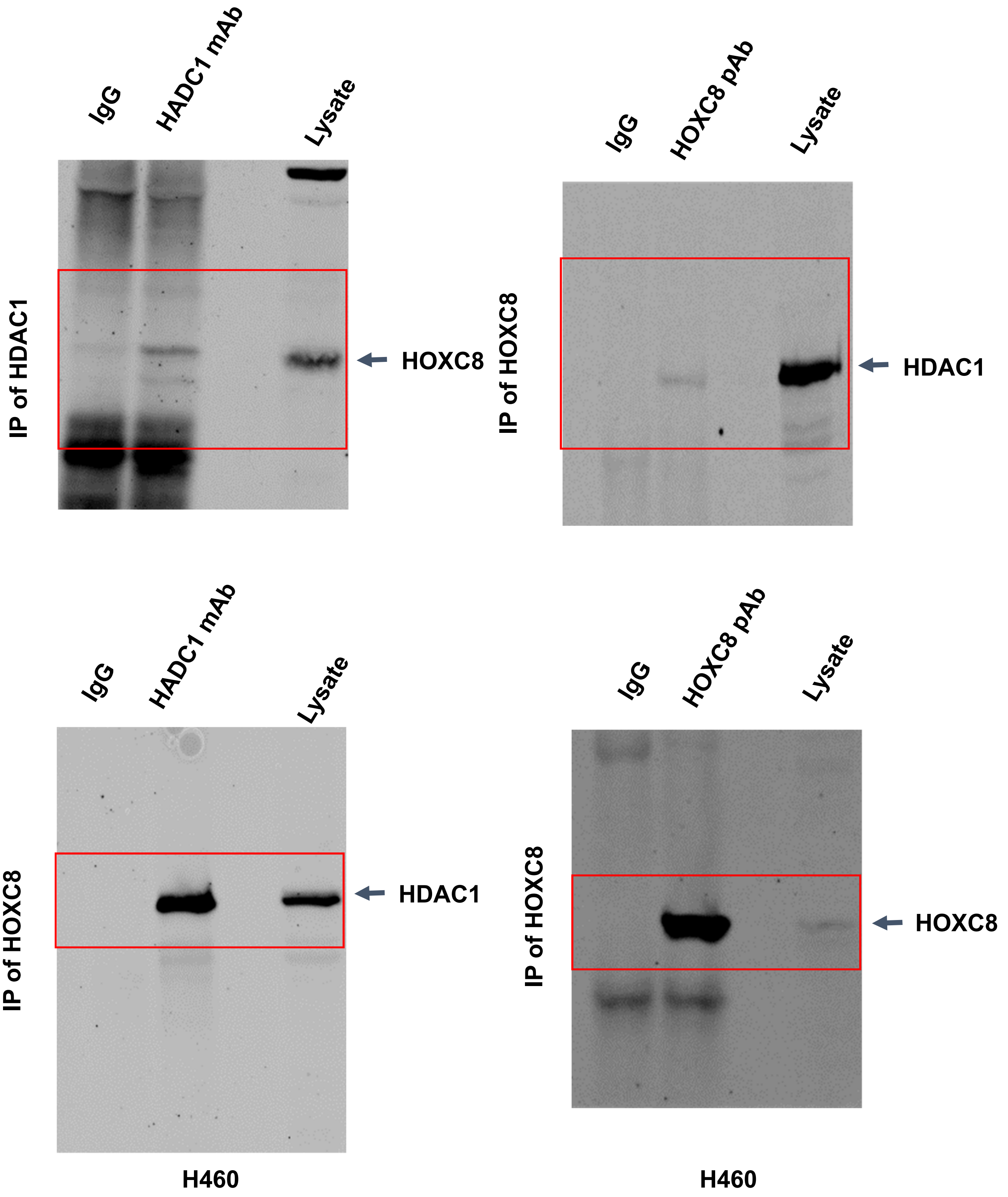
IgG
HADC1 mAb
Lysate
IP of HDAC1
HOXC8
IgG
HOXC8 pAb
Lysate
IP of HOXC8
HDAC1
IgG
HADC1 mAb
Lysate
IP of HOXC8
HDAC1
IgG
HOXC8 pAb
Lysate
IP of HOXC8
HOXC8
H460
H460

Figure 6D

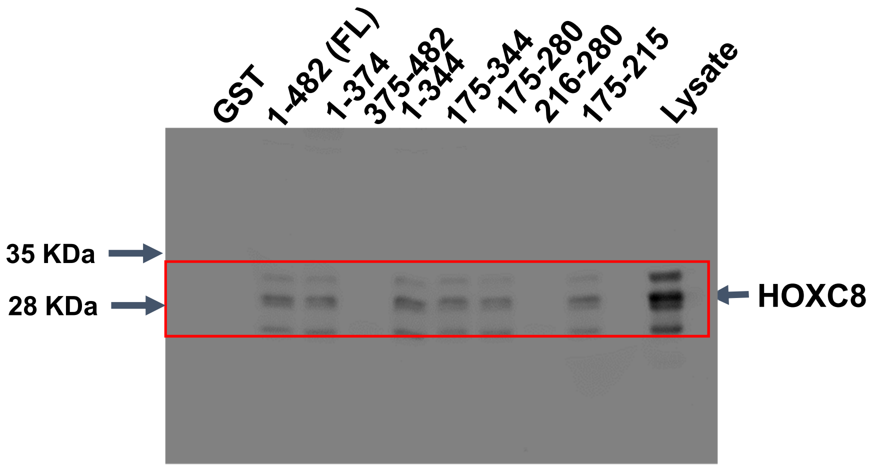
GST
1-482 (FL)
1-374
375-482
1-344
175-344
175-280
216-280
175-215
Lysate
35 KDa
28 KDa
HOXC8

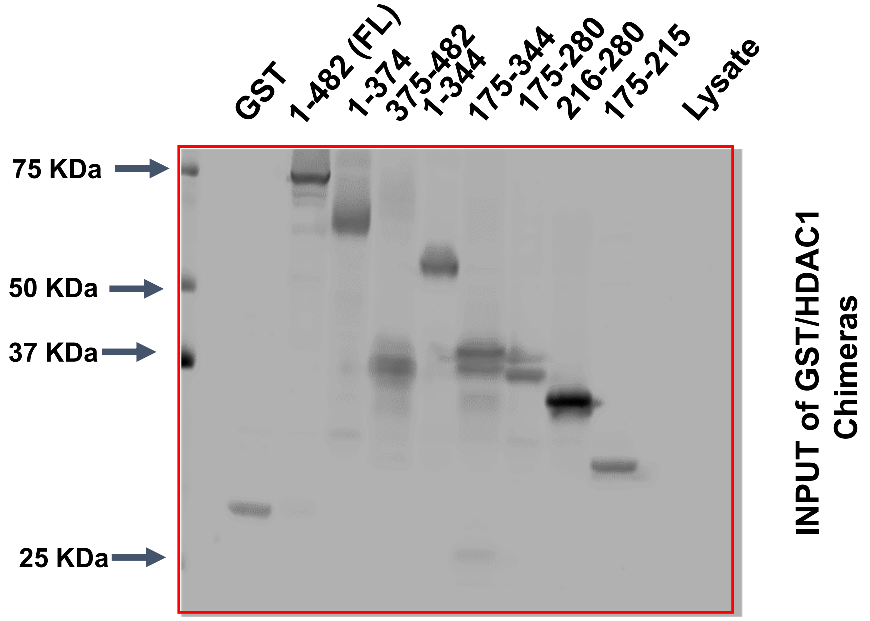
GST
1-482 (FL)
1-374
375-482
1-344
175-344
175-280
216-280
175-215
Lysate
75 KDa
50 KDa
37 KDa
25 KDa
INPUT of GST/HDAC1
Chimeras

Supplemental Figure S3

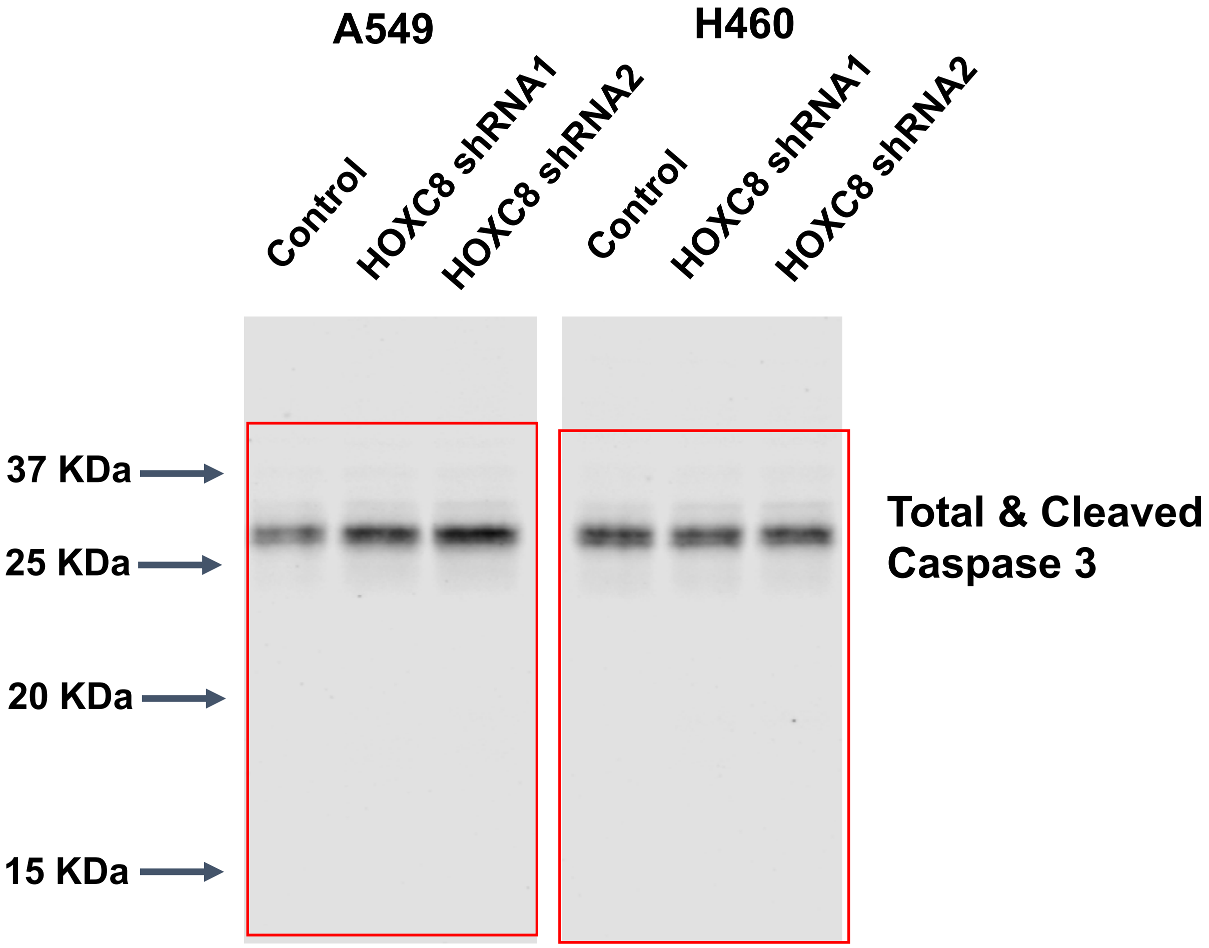

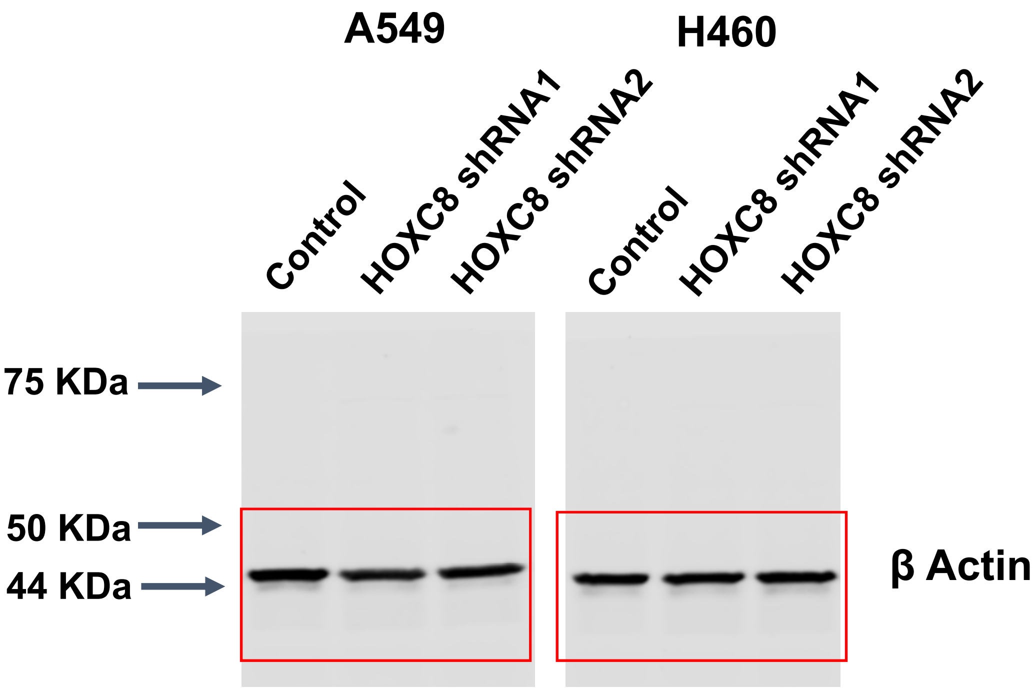

Supplemental Figure S6

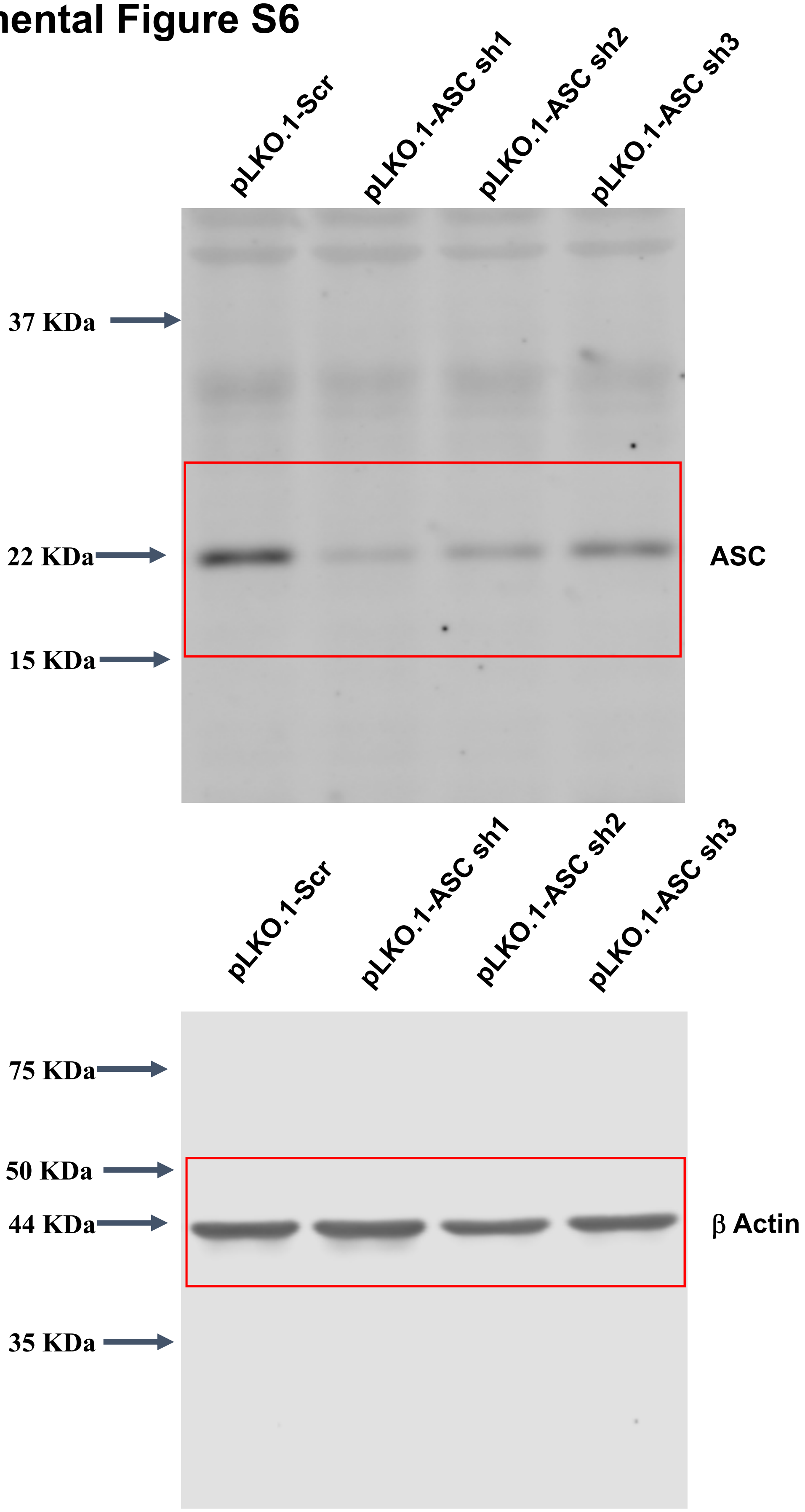

Supplemental Figure S8A

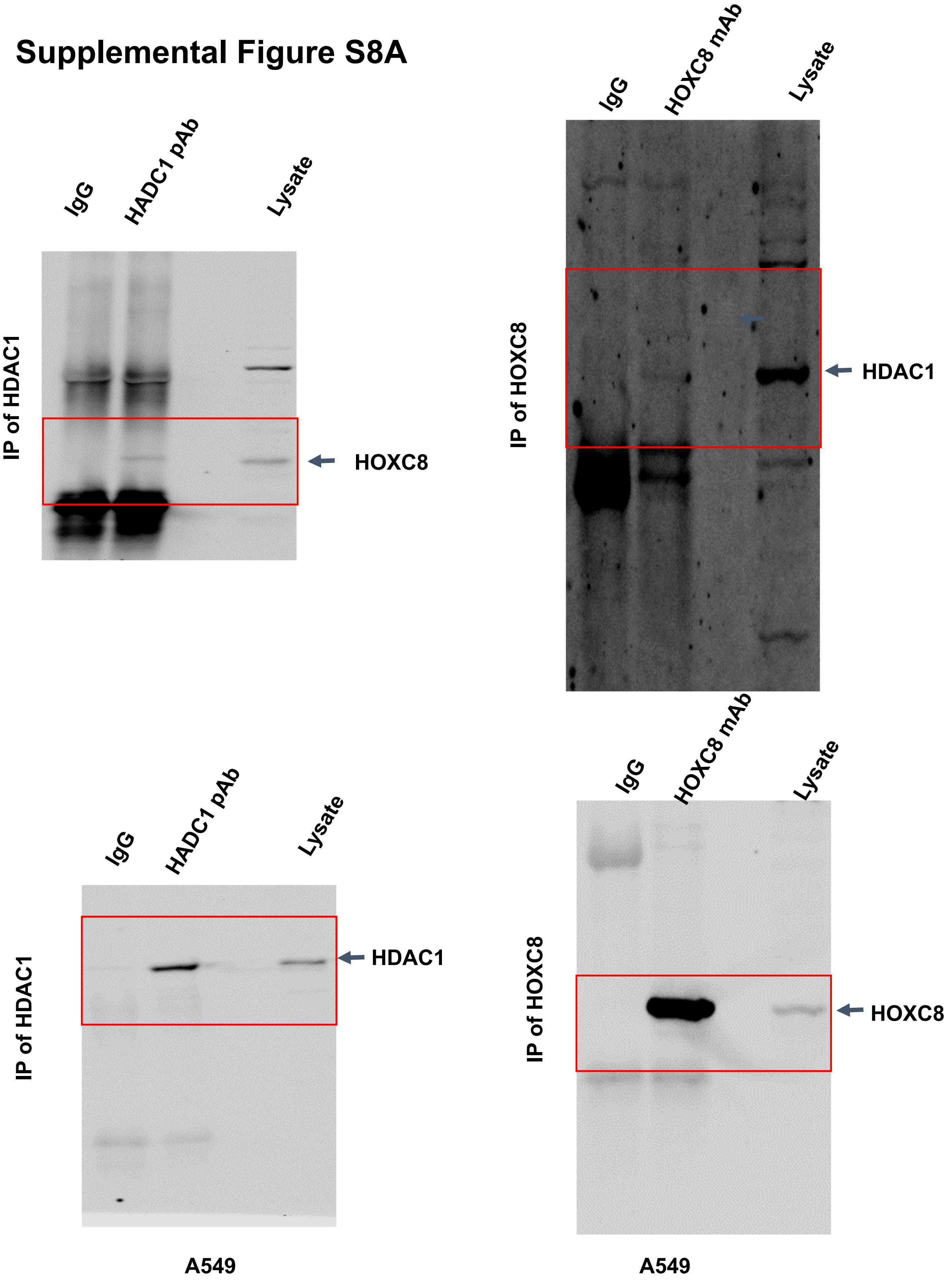

Supplement: Supplementary file 2 — Uncropped gels [file 41419_2025_7867_MOESM2_ESM.pdf]
